# Multimodal GPT-5 for Predicting Poor Functional Outcomes After Intracerebral Hemorrhage in the Emergency Department: Validation Study

**DOI:** 10.2196/87062

**Published:** 2026-05-27

**Authors:** Koutarou Matsumoto, Kazuaki Ishihara, Ryota Tamba, Yusuke Fujiyoshi, Koki Tokunaga, Katsuhiko Matsuda, Yasunobu Nohara, Jenhui Chen, Shigeo Yamashiro, Naoki Nakashima, Masahiro Kamouchi

**Affiliations:** 1 Department of Health Care Administration and Management, Graduate School of Medical Sciences, Kyushu University, 3-1-1 Maidashi, Higashi-ku, Fukuoka-shi, Fukuoka, 812-8582, Japan, 81 0926426960; 2 Institute for Medical Information Research and Analysis, Saiseikai Kumamoto Hospital, Kumamoto, Japan; 3 Department of Computer Science and Information Engineering, Chang Gung University, Taoyuan, Taiwan; 4 Graduate Degree Program of Applied Data Sciences, Sophia University, Tokyo, Japan; 5 Joint Graduate School of Mathematics for Innovation, Kyushu University, Fukuoka, Japan; 6 Department of Pharmacy, Saiseikai Kumamoto Hospital, Kumamoto, Japan; 7 Department of Radiology, Saiseikai Kumamoto Hospital, Kumamoto, Japan; 8 Big Data Science and Technology, Faculty of Advanced Science and Technology, Kumamoto University, Kumamoto, Japan; 9 Division of Neurosurgery, Saiseikai Kumamoto Hospital, Japan; 10 Medical Information Center, Kyushu University Hospital, Fukuoka, Japan; 11 Department of Medical Informatics, Graduate School of Medical Sciences, Kyushu University, Fukuoka, Japan; 12 Center for Cohort Studies, Graduate School of Medical Sciences, Kyushu University, Fukuoka, Japan

**Keywords:** multimodal model, zero-shot learning, generative pretrained transformer, GPT, intracerebral hemorrhage, generative artificial intelligence, machine learning

## Abstract

**Background:**

In the emergency department, rapid prognostic assessment of patients with intracerebral hemorrhage (ICH) is essential for guiding early management decisions, particularly when stroke specialists are not immediately available. Recent advances in large language models have generated interest in their potential utility as clinical decision-support tools.

**Objective:**

This study aimed to evaluate the predictive performance and potential clinical utility of GPT (OpenAI)-based models for poor functional outcomes after ICH using real-world multimodal data routinely available at emergency department presentation.

**Methods:**

We analyzed data from patients with ICH admitted to a tertiary hospital. Using routinely collected clinical data and noncontrast computed tomography (CT) images at admission, GPT-4.1 (OpenAI) and GPT-5 (OpenAI)—accessed via the Azure OpenAI Service—were applied to predict poor functional outcomes, defined as a modified Rankin Scale score of 3‐6 at discharge. A conventional machine learning (ML) model was developed by combining deep learning–extracted imaging features from Digital Imaging and Communications in Medicine CT data with clinical variables using L1-regularized logistic regression. GPT-based models were evaluated using the same clinical dataset and JPEG-format CT images. Model performance was assessed in terms of discrimination (area under the receiver operating characteristic curve [AUROC]), overall performance (scaled Brier score and Nagelkerke *R*²), calibration, reproducibility (intraclass correlation coefficient [ICC]), and clinical utility (decision curve analysis).

**Results:**

The ML model achieved an AUROC of 0.85 (95% CI 0.79‐0.90), a scaled Brier score of 0.23 (95% CI 0.06‐0.36), and a Nagelkerke *R*² of 0.35 (95% CI 0.18‐0.48). Zero-shot GPT-4.1 and GPT-5 demonstrated discrimination comparable to the ML model (AUROC 0.84, 95% CI 0.77‐0.91 and 0.85, 95% CI 0.78‐0.91, respectively) with high reproducibility (ICC 0.91 and 0.95, respectively) but inferior overall performance, as reflected by lower scaled Brier scores and low or negative Nagelkerke *R*² values. Incorporating ML-derived information into the prompts modestly improved discrimination (AUROC 0.84, 95% CI 0.78‐0.90 and 0.87, 95% CI 0.81‐0.92, respectively) and reproducibility (ICC 0.97 and 0.96, respectively). Calibration plots indicated that GPT-based models tended to underestimate predicted probabilities, although this bias was partially attenuated after model-informed prompting. Decision curve analysis indicated that GPT-based models provided net benefit only at higher threshold probabilities and did not demonstrate superior clinical utility compared with the ML model.

**Conclusions:**

Zero-shot GPT models achieved discriminatory performance comparable to a conventional ML model but showed limitations in calibration and overall predictive accuracy. Rather than replacing established prognostic ML models, GPT-based models may be better positioned as complementary interfaces that translate predictive outputs into clinically interpretable natural language to support decision-making.

## Introduction

Intracerebral hemorrhage (ICH) accounts for approximately 10%‐30% of all strokes yet carries disproportionately high morbidity and mortality rates [1,2]. It is a life-threatening neurological emergency that demands prompt and accurate decision-making upon hospital arrival. ICH onset is unpredictable; therefore, stroke specialists are not always available in the emergency department (ED). Therefore, establishing systems that support rapid decision-making by nonspecialists and enable seamless handover to stroke specialists is essential.

In routine clinical practice, emergency physicians first gather initial clinical information, conduct brain imaging, and consult a stroke specialist once ICH is confirmed. Although nonspecialists can usually recognize ICH on imaging, accurately estimating a patient’s functional prognosis, which is crucial for determining the treatment strategy, remains challenging. Effective clinical reasoning in this setting requires integrating multimodal information, including imaging, vital signs, laboratory data, and medical history, into a coherent assessment.

Recent advances in deep learning have enabled multimodal algorithms that predict ICH prognosis early after presentation [3-6]. More recently, large language models (LLMs) have emerged with the capability to process tabular, textual, and imaging inputs. As these models have evolved, foundation models fine-tuned with medical data have been increasingly adapted for diverse health care applications [7]. In parallel, closed-source general-purpose LLMs, including OpenAI’s GPT, Google Gemini, and Anthropic Claude, have shown strong performance on complex reasoning tasks even without task-specific training.

Released in August 2025, GPT-5 represents one of the most advanced LLMs to date [8]. Early studies have indicated that GPT-5 can integrate multimodal inputs, including medical images and structured data, to perform sophisticated clinical reasoning and achieve high accuracy on standardized multimodal benchmarks [9-11]. However, despite these advances, its prognostic reasoning performance with real-world clinical data has not been evaluated, and no prior study has validated its use for predicting functional outcomes in patients with ICH.

Therefore, in this study, from the perspective of emergency physicians, we evaluated how accurately GPT models predict poor functional outcomes—herein defined as a modified Rankin Scale (mRS) score ≥3—after ICH using only routinely available ED clinical data and brain images converted to compact JPEG files suitable for remote transmission. We compared the performance of GPT with that of a conventional machine learning (ML) model trained on the same clinical variables and image features extracted from Digital Imaging and Communications in Medicine (DICOM) computed tomography (CT) scans via deep learning. Our specific objectives were to assess the zero-shot predictive performance of GPT-4.1 and GPT-5 using ED clinical data and brain imaging without any additional fine-tuning and determine whether incorporating outputs from a conventional ML model could further enhance GPT performance.

## Methods

### Ethical Considerations

The study protocol was approved by the Ethics Committee of Saiseikai Kumamoto Hospital (approval number 1199; September 29, 2023) and conducted in accordance with the Declaration of Helsinki. All data were fully anonymized before analysis and retrospectively analyzed; therefore, the requirement for informed consent was formally waived by the institutional Ethics Committee. No compensation was provided to study participants. The paper and supplementary materials include only noncontrast head CT images that do not permit identification of individual participants. Images shown in Multimedia Appendix 1 were obtained from a publicly available anonymized dataset [12].

### Study Design and Participants

Validation and reporting of GPT-based predictions followed the Transparent Reporting of a Multivariable Model for Individual Prognosis or Diagnosis (TRIPOD)-LLM statement [13]. To compare the performance of GPT-based predictions with that of the ML model, we used the same datasets as in our previous studies [3,4], which developed multimodal ML-based models for predicting poststroke unfavorable discharge outcomes, including poor functional outcome and in-hospital mortality. In the previous analysis, 527 patients with ICH were included. Among them, the derivation cohort comprised 352 patients admitted between April 2019 and December 2020, and the temporal validation cohort included 175 patients admitted between January 2021 and January 2022 (Multimedia Appendix 2). In this study, all comparisons and validations of GPT and ML model performance were conducted using this temporal validation cohort.

### Clinical Outcome

A poor functional outcome was defined as an mRS score of 3‐6 at hospital discharge. The mRS score was assessed by the attending physician.

### Tabular Data

Clinical data comprised tabular variables combined with noncontrast head CT images that were routinely obtainable by nonspecialists at ED presentation. A detailed list of the tabular variables used in this study, together with their corresponding missing rates, is provided in Table 1. Although multiple imputation is generally recommended, directly applying multiple imputation within a GPT-based inference framework is technically challenging. In addition, the proportion of missing values across all variables in this study was low (maximum <3.5%), indicating a limited loss of information and an unlikely material impact on the results [14]. Therefore, single imputation was performed using the Multivariate Imputation by Chained Equations method implemented in the *mice* package in R. To prevent data leakage, missing value imputation was performed after splitting the dataset into the derivation and temporal validation cohorts and was conducted independently within each cohort [3,4].

**Table 1.** Tabular clinical variables used for both GPT zero-shot inference and ML^a^-based model^b^.

Variable	Summary statistics	Missing rate
Demographic		
Age (y), median (IQR)	75 (64‐83)	0.0
Female, n (%)	69 (39.4)	0.0
Risk factor, n (%)		
Hypertension	126 (72.0)	3.4
Diabetes mellitus	41 (23.4)	3.4
Dyslipidemia	58 (33.1)	3.4
Smoking	60 (34.3)	0.6
Drinking	72 (41.1)	2.9
Prestroke functional status, median (IQR)		
Prestroke mRS^c^ score	0 (0‐1)	0.6
Prestroke medication, n (%)		
Antiplatelets	40 (22.9)	3.4
Direct oral anticoagulants	24 (13.7)	3.4
Vitamin K antagonists	6 (3.4)	3.4
Onset-to-admission time, n (%)		
<4 h	91 (52.0)	0.0
4‐8 h	18 (10.3)	0.0
8‐24 h	39 (22.3)	0.0
24‐72 h	24 (13.7)	0.0
>72 h	3 (1.7)	0.0
Transportation method to the hospital, n (%)		
Ambulance use	161 (92.0)	0.0
Physiological data, median (IQR)		
Systolic blood pressure (mm Hg)	180 (162.5‐202)	0.0
Diastolic blood pressure (mm Hg)	106 (93.5‐121)	0.0
Pulse rate (per min)	83 (70‐95.5)	0.0
SpO_2_ (%)	97 (95‐98)	0.0
Respiratory rate (per min)	19 (17‐22)	0.0
Body temperature (℃)	36.6 (36.4‐36.9)	1.7
BMI (kg/m²)	22.9 (19.5‐25.6)	1.1
Laboratory data, median (IQR)		
Sodium (mmol/L)	141 (139‐142)	0.0
Potassium (mmol/L)	3.8 (3.6‐4.2)	0.0
Chloride (mmol/L)	104 (101‐105)	0.0
Total protein (g/dL)	7.1 (6.75‐7.55)	0.0
Albumin (g/dL)	4.0 (3.8‐4.3)	0.0
Blood urea nitrogen (mg/dL)	16.1 (13.65‐19.75)	0.0
Creatinine (mg/dL)	0.82 (0.67‐1.04)	0.0
Aspartate aminotransferase (IU/L)	25 (20‐32.5)	0.0
Alanine transaminase (IU/L)	17 (13.5‐25.5)	0.0
Gamma-glutamyl transferase (IU/L)	25 (15‐48.5)	0.0
Lactate dehydrogenase (IU/L)	224 (195.5‐263)	0.0
Total bilirubin (mg/dL)	0.8 (0.6‐1.0)	0.0
Glucose (mg/dL)	127 (108‐161.5)	0.0
White blood cell count (10^3^/μL)	7.60 (6.15‐10.10)	0.0
Red blood cell count (10^6^/μL)	4.45 (3.96‐4.88)	0.0
Hemoglobin (g/dL)	13.7 (12.4‐15.05)	0.0
Hematocrit (%)	40.8 (37.2‐44.5)	0.0
Blood platelet count (10^4^/μL)	21.2 (17.05‐24.6)	0.0
C-reactive protein (mg/dL)	0.10 (0.05‐0.26)	0.0
Activated partial thromboplastin time (s)	27.6 (25.4‐30.4)	0.0
Prothrombin time-international normalized ratio, median (IQR)	0.97 (0.91‐1.03)	0.6

a ML: machine learning.

b Data are expressed as median (IQR) or n (%). The rates of missing values (%) are shown for clinical variables.

c mRS: modified Rankin Scale.

### Image Data

CT images were acquired using a Canon Aquilion Prime SP scanner (Canon Medical Systems Corporation) and stored in DICOM format within the hospital’s picture archiving and communication system (Multimedia Appendix 3). The image preprocessing procedure for the ML model was identical to that described in our previous report [4]. Briefly, noncontrast CT images were adjusted to a window range of 15‐100 Hounsfield units, followed by min-max normalization. After centering the brain, each image was resized to 256×256 pixels. The number of axial slices was standardized to 22 using spline interpolation, as specified in the study protocol.

In contrast, for the GPT models, CT images in JPEG format were used as model inputs. Representative slices that clearly demonstrated ICH were used owing to the limit of 10 images per chat call when uploading images through the application programming interface (API). For each patient, an experienced radiologic technologist, blinded to discharge mRS scores and all baseline clinical admission data used in the prediction models, selected slices deemed clinically relevant for diagnostic reporting according to predefined criteria: images including the hematoma; images capturing the overall hematoma when large—covering the upper margin, central portion (multiple slices if necessary), and lower margin; and images showing intraventricular extension or midline shift when present. The selected images (median 3, IQR 2‐4 slices; Multimedia Appendix 4) were converted from DICOM to compact JPEG format to emulate real-world ED workflows, where nonspecialists frequently share lightweight image files for remote consultation. These JPEG images were used to facilitate standardized multimodal input and efficient remote processing during GPT inference.

This design enabled a direct comparison between a conventional ML model trained on fully standardized DICOM images and GPT models operating on simplified, nonspecialist-accessible JPEG images, thereby reflecting distinct yet complementary clinical use cases.

### GPT Configuration

GPT-5 (version 2025-08-07) was accessed via the Azure OpenAI Service using the Responses API [15], which supports multimodal input. The model had been trained on data updated until September 2024. Based on preliminary analyses showing negligible differences in predictive accuracy between the minimal and high reasoning-effort configurations (internally defined by Azure OpenAI), the reasoning effort was fixed at the minimal level for all experiments (Multimedia Appendix 5). The verbosity level (response length control) was set to low to reduce unnecessary variation and ensure concise, reproducible outputs.

The maximum token limit was not explicitly specified; the provider’s default setting was used because all clinical summaries were concise and remained well within this limit. To ensure complete independence of inference across patients, conversation history and system context were cleared before each request, and each case was separately processed. All GPT-5 inference experiments were conducted between August 17 and 23, 2025.

For comparison, GPT-4.1 (version 2025-04-14) was also evaluated via the Azure OpenAI Service using the Chat Completions API [15]. This model had been trained on data updated until April 2024. Unlike GPT-5, the Chat Completions API allowed explicit control of the temperature parameter; therefore, the temperature was set to 0 to minimize sampling variability and improve output consistency across runs. Nevertheless, exact reproducibility may not always be achieved, as closed-source LLMs may exhibit nondeterministic behavior due to factors such as floating-point operations and distributed inference. All GPT-4.1 inference experiments were performed during the same period as GPT-5 (August 17‐23, 2025).

### ML-Based Model

For comparison with GPT, we used an ML-based model previously developed by our group, with minor modifications where the tabular clinical inputs were replaced with the same variables used for GPT inference (Table 1). This model adopted a late-fusion architecture that combined imaging features extracted from noncontrast head CT scans with structured tabular variables obtainable by nonspecialists, as listed in Table 1.

Imaging features were extracted using a 3D U-Net pretrained on the publicly available Brain Hemorrhage Segmentation Dataset for ICH segmentation [12]. Fully connected layers were added to the encoder output and subsequently fine-tuned using 3D CT data from Saiseikai Kumamoto Hospital to predict discharge outcomes (mRS score ≥3) based on binary cross-entropy loss. This fine-tuning process allowed the pretrained encoder to adapt its high-level feature representations to the target classification task while capturing local imaging characteristics specific to the institutional dataset.

From the fine-tuned network, a 512-dimensional feature vector was obtained via a global average pooling layer applied to the final encoder output. This vector was concatenated with standardized tabular variables, and an L1-regularized logistic regression model was trained to construct the final late-fusion model. The use of L1 regularization promoted sparsity and facilitated feature selection among the combined multimodal inputs. Model training and fine-tuning were performed on the derivation cohort (n=352), and model performance was assessed on the same independent temporal validation cohort (n=175) as that used for GPT evaluation.

### Prompt Design and GPT Inference

**Figure 1.**
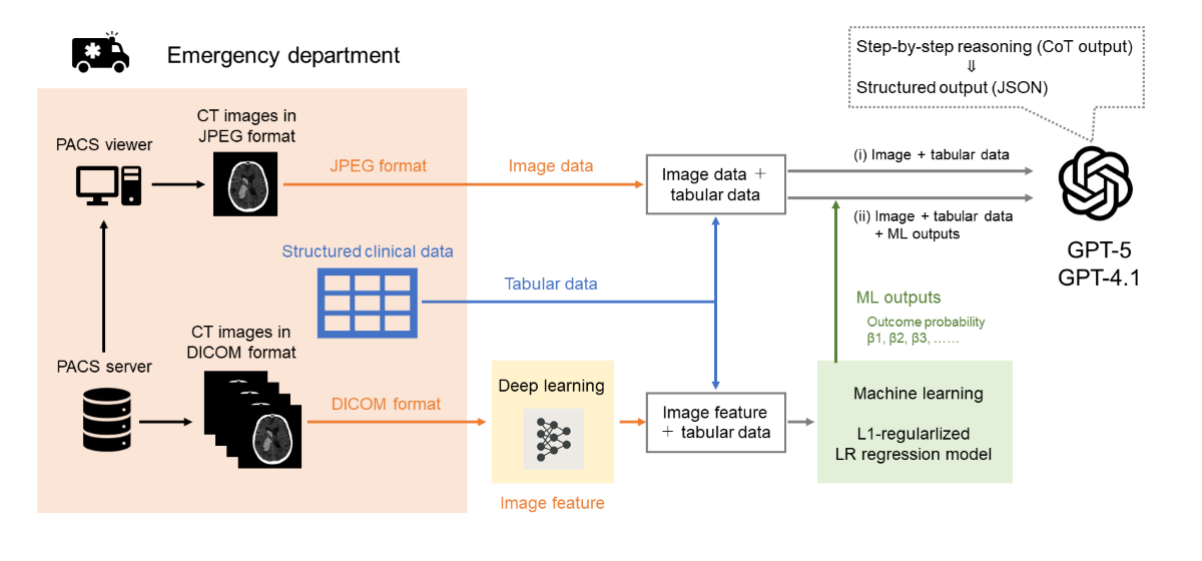
Overview of GPT-based zero-shot inference and the machine learning–assisted prompting workflow. CoT: chain-of-thought output; CT: computed tomography; DICOM: Digital Imaging and Communications in Medicine; JSON: JavaScript Object Notation; LR: logistic regression; ML: machine learning; PACS: Picture Archiving and Communication System.

Figure 1 summarizes the 2 types of prompt strategies used in this study. Detailed examples of each prompt type are provided in Multimedia Appendix 6 (Prompts S1 and S2). (1) Tabular-image integration prompt: structured tabular variables were combined with noncontrast head CT images selected by a radiologic technologist. The GPT model was instructed to jointly process both modalities and integrate image-derived features with clinical data during reasoning (Prompt S1).

(2) Model-informed prompt: outputs from the ML-based model described above—including predicted probabilities and standardized regression coefficients (Multimedia Appendix 7)—were appended to the GPT input. This design allowed GPT to incorporate prior model knowledge, including feature importance, together with patient-specific tabular and imaging data during inference (Prompt S2). The predicted probabilities were provided to support calibration of the GPT-generated probabilities, whereas the standardized regression coefficients were included to explicitly convey the direction of risk contribution for each feature, thereby improving clinical interpretability. Because the predicted probabilities already incorporate information from the model intercept, the intercept itself was not explicitly included.

Across both prompt types, GPT generated quantitative predictions of the probability of a poor functional outcome (discharge mRS score ≥3) expressed as a percent (0%‐100%), accompanied by a concise explanation of its reasoning process. Representing the outcome as a continuous probability enabled quantitative evaluation of both discrimination and calibration. To enhance interpretability, a zero-shot chain-of-thought prompting approach was used, instructing the model to generate a brief reasoning statement before providing the final probability estimate [16]. All outputs were formatted in a unified JavaScript Object Notation structure to ensure consistency across cases and facilitate automated postprocessing and evaluation of performance metrics.

As a preliminary test of model behavior before applying real patient data, we created synthetic clinical data representing hypothetical patients and asked the GPT models to estimate prognostic outcomes. A stroke specialist (MK) designed these pseudopatient profiles using the same variable structure as the real dataset from Saiseikai Kumamoto Hospital, representing relatively low-risk clinical scenarios (eg, mild neurological severity and smaller hematoma volume). For demonstration purposes, the synthetic cases were paired with brain CT images from the publicly available Brain Hemorrhage Segmentation Dataset [12].

### Performance Evaluation

The discriminative performance of GPT-based predictions was evaluated using the area under the receiver operating characteristic curve (AUROC), sensitivity, specificity, positive predictive value (PPV), and negative predictive value (NPV). Sensitivity, specificity, the PPV, and the NPV were calculated at the optimal cutoff determined by the Youden index. In this study, the scaled Brier score and Nagelkerke *R*² were calculated as overall predictive accuracy measures. The scaled Brier score was defined as the improvement in the Brier score relative to a noninformative null model that assigns the overall outcome incidence as a constant predicted probability to all individuals. Nagelkerke *R*² was calculated using the same null model based on the improvement in log-likelihood. Model calibration was visually assessed using calibration plots. Calibration curves were generated using Locally Estimated Scatterplot Smoothing (LOESS) with a span parameter of 0.80.

The ML-based model was evaluated using the same temporal cohort as that used for GPT. For the ML-based and GPT-based models, 95% CIs were estimated using 2000 bootstrap resamples based on predicted probabilities from a single representative GPT inference run per patient to assess the stability of the performance metrics. To evaluate reproducibility, GPT inference was repeated 5 times for each case under identical conditions, and the consistency of predicted probabilities across runs was quantified using the intraclass correlation coefficient (ICC; single-score, 1-way random-effects model). Finally, decision curve analysis (DCA) was conducted to assess clinical utility by calculating the net benefit—defined as the trade-off between true-positive and false-positive predictions weighted by the relative harm of false-positives—across varying probability thresholds derived from GPT predictions [17,18]. Decision curves were smoothed using LOESS with a span parameter of 0.20 to reduce variability in the plotted net benefit curves.

### Image Contribution and Validity of GPT-Estimated Hematoma Volume

To evaluate the contribution of image inputs to GPT-based inference, we conducted an ablation analysis. Specifically, inference using structured tabular clinical data alone was compared with inference using both noncontrast head CT images and tabular data in terms of discriminative performance. To assess the validity of image-derived outputs generated by GPT, we examined the agreement between GPT-estimated hematoma volume and imaging-based reference standards. The measured hematoma volume was used as the reference standard and was calculated using the SYNAPSE VINCENT 3D image analysis system (Fujifilm Medical) by the same radiologic technologist who selected the representative JPEG slices and remained blinded to discharge mRS scores and baseline clinical admission data. GPT-based hematoma volume estimation was performed using the ABC/2 method, and the association between GPT-estimated and measured hematoma volumes was evaluated using Spearman rank correlation coefficient.

### Sensitivity Analysis

To evaluate the impact of a good premorbid functional status, a sensitivity analysis was conducted restricting the cohort to patients who were functionally independent at baseline (premorbid mRS 0‐1), consistent with common practice in stroke research. The same models and evaluation procedures used in the primary analysis were applied.

### Software

Prompt generation and inference using GPT via the Azure OpenAI API were implemented in Python (version 3.13.7; Python Software Foundation). The ML experiments were conducted in Python (version 3.10.11; Python Software Foundation) using PyTorch (version 1.11.0; Meta Platforms). Evaluation of predictive performance and ICC was performed with RStudio (version 2025.05.0; Posit) in R statistical software (version 4.5.0; R Foundation for Statistical Computing). All codes and implementation details are available in the GitHub repository referenced in Multimedia Appendix 8.

## Results

### Patient Characteristics

Among the 175 patients in the temporal validation cohort, 139 (79.4%) had poor functional outcomes (mRS score=3‐6) at discharge, including those who died (mRS score=6). The distributions and missing rates of tabular variables used as model inputs for the GPT and ML are summarized in Table 1.

### Preliminary Test of Model Behavior

The results of GPT-based prognostic inference using synthetic clinical data representing pseudopatients are shown in Multimedia Appendix 1. In this experiment, 2 different CT images were provided as input predictors for the same set of clinical variables. The structured tabular inputs were identical between the 2 cases, whereas the CT images differed in hematoma volume (A: small hematoma; B: large hematoma). GPT-5 produced different prognostic estimates according to hematoma size, assigning a higher risk to the large-hematoma case (B), confirming that the model’s outputs appropriately varied in response to image-based prognostic cues.

### Predictive Performance

The predictive performance of the clinical risk score, the conventional ML-based model, and the GPT-based models is summarized in Table 2. For reference, the performance of the established FUNC score, previously validated in the same cohort in our prior study [4], is also reported in Table 2. Overall, both the conventional ML-based model and the GPT-based models showed discriminative performance comparable to that of the FUNC score. The conventional ML-based model achieved an AUROC of 0.85 (95% CI 0.79‐0.90), along with high specificity and PPV. The scaled Brier score for the ML model was 0.23 (95% CI 0.06‐0.36), with a corresponding Nagelkerke *R*² of 0.35 (95% CI 0.18‐0.48), indicating good overall predictive performance. Among the zero-shot models, GPT-4.1 and GPT-5 achieved AUROCs of 0.84 (95% CI 0.77-0.91) and 0.85 (95% CI 0.78-0.91), respectively, indicating discrimination similar to that of the conventional ML-based model. However, the overall predictive performance of zero-shot GPT-5 was inferior to that of the null model. The scaled Brier score for GPT-5 was low and included negative values (−0.17; 95% CI −0.58 to 0.10); Nagelkerke *R*² was also negative (−0.16; 95% CI −0.67 to 0.14). Negative Nagelkerke *R*² values can occur when the model’s predicted probabilities produce a log-likelihood worse than that of a noninformative null model, reflecting miscalibration of the probability estimates. For the ML-assisted GPT-4.1 model, AUROC remained largely unchanged, although sensitivity increased relative to the zero-shot GPT-4.1 model. In contrast, the ML-assisted GPT-5 model demonstrated the highest discrimination, with an AUROC of 0.87 (95% CI 0.81-0.92). This model also achieved high specificity and PPV. The scaled Brier score for this model was improved to 0.19 (95% CI −0.10 to 0.36), and Nagelkerke *R*² was increased to 0.29 (95% CI −0.001 to 0.46), suggesting partial improvement in overall predictive performance, although not surpassing that of the stand-alone ML model. The probability thresholds derived using the Youden method were consistently higher for the ML-assisted models than for their zero-shot counterparts. Specifically, the threshold increased from 0.68 (95% CI 0.50-0.73) to 0.82 (95% CI 0.61-0.91) for GPT-4.1 and from 0.46 (95% CI 0.37-0.52) to 0.57 (95% CI 0.50-0.73) for GPT-5. The reproducibility of GPT-based predictions across 5 repeated inference runs is summarized in Multimedia Appendix 9. Both GPT-4.1 and GPT-5 demonstrated high interrun agreement, which was further enhanced by model-informed prompting.

**Table 2.** Predictive performance of GPT-based models, an ML^a^-based model, and a clinical risk score.

Risk score or model	AUROC^b^	Sensitivity	Specificity	PPV^c^	NPV^d^	Scaled BS^e^	Nagelkerke *R*²
Risk score, estimated performance metrics (95% CI)							
FUNC score	0.80 (0.73-0.86)	0.61 (0.53-0.70)	0.94 (0.86-1.00)	0.98 (0.94-1.00)	0.39 (0.34-0.45)		
Model type, estimated performance metrics (95% CI)							
ML-based model	0.85 (0.79-0.90)	0.72 (0.62-0.85)	0.94 (0.81-1.00)	0.98 (0.94-1.00)	0.47 (0.39-0.59)	0.23 (0.06-0.36)	0.35 (0.18-0.48)
Zero-shot model, estimated performance metrics (95% CI)							
GPT-4.1	0.84 (0.77-0.91)	0.65 (0.54-0.89)	0.92 (0.70-1.00)	0.97 (0.91-1.00)	0.41 (0.32-0.66)	0.13 (–0.15 to 0.33)	0.21 (−0.11 to 0.42)
GPT-5	0.85 (0.78-0.91)	0.68 (0.58-0.85)	0.92 (0.76-1.00)	0.97 (0.92-1.00)	0.42 (0.32-0.60)	–0.17 (–0.58 to 0.10)	−0.16 (−0.67 to 0.14)
ML-assisted model, estimated performance metrics (95% CI)							
GPT-4.1-assisted by ML	0.84 (0.78-0.90)	0.71 (0.62-0.85)	0.92 (0.77-1.00)	0.97 (0.93-1.00)	0.45 (0.34-0.59)	0.22 (0.02-0.37)	0.34 (0.14-0.48)
GPT-5-assisted by ML	0.87 (0.81-0.92)	0.72 (0.61-0.82)	0.94 (0.87-1.00)	0.98 (0.96-1.00)	0.47 (0.34-0.59)	0.19 (–0.10 to 0.36)	0.29 (−0.001 to 0.46)

a ML: machine learning.

b AUROC: area under the receiver operating characteristic curve.

c PPV: positive predictive value.

d NPV: negative predictive value.

e BS: Brier score.

Values are presented as point estimates with 95% CIs in parentheses. All 95% CIs were estimated using 2000 bootstrap resamples based on predicted probabilities from a single representative inference run.

**Figure 2.**
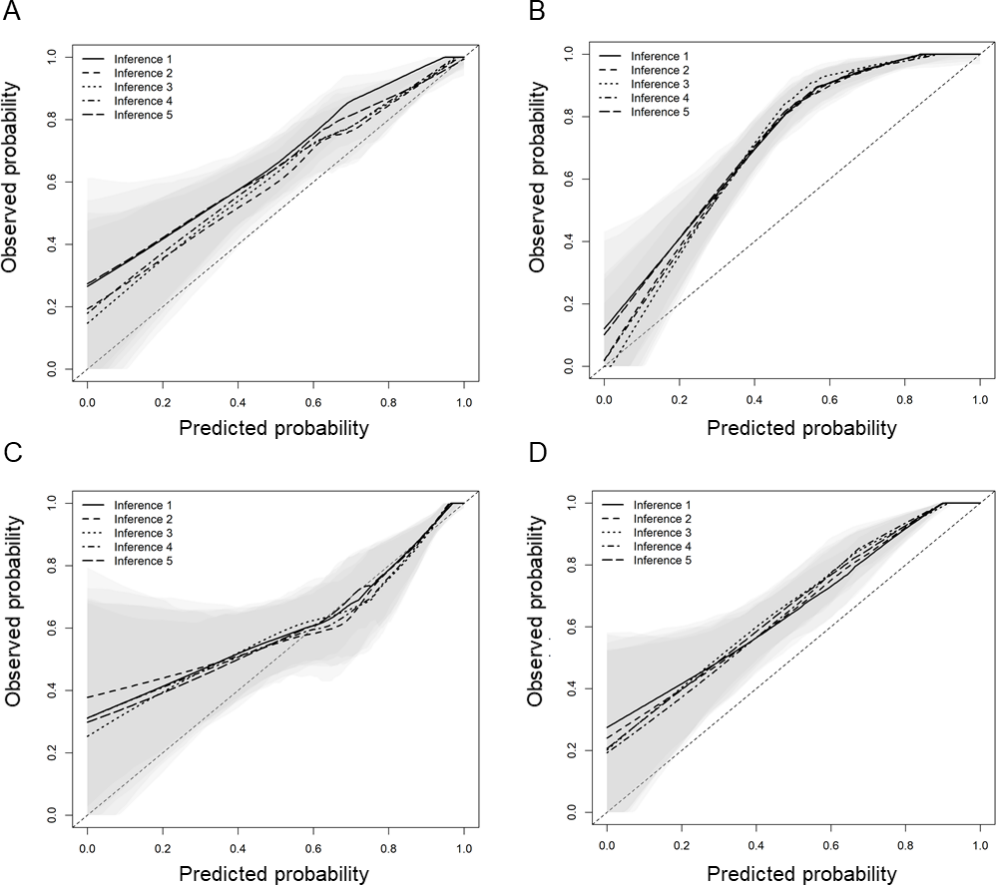
Calibration of GPT-4.1 and GPT-5 with and without model-informed prompting.

Calibration plots for the ML model are shown in Multimedia Appendix 10, and those for the 5 repeated inference runs are presented in Figure 2. Both GPT-4.1 and GPT-5 initially tended to underestimate predicted probabilities, as indicated by calibration curves lying above the ideal diagonal line. After model-informed prompting, this bias appeared to be partially attenuated, with a tendency toward closer agreement between the observed and predicted probabilities across risk levels. The improvement was more apparent for GPT-5 but modest and primarily confined to the higher-risk range for GPT-4.1. Taken together, these findings suggest that model-informed prompting may influence discrimination, reproducibility, and calibration; however, it does not fully address the limitations in predictive reliability.

Calibration plots for (A) GPT-4.1, (B) GPT-5, (C) model-informed GPT-4.1, and (D) model-informed GPT-5 in the validation cohort. The relationship between predicted probabilities (*x*-axis) and observed probabilities (*y*-axis) is shown using a LOESS-smoothed calibration curve. The shaded area represents the pointwise 95% CI estimated by patient-level bootstrap resampling. Predicted probabilities from 5 independent inference runs are overlaid to illustrate interrun variability and reproducibility. The dashed diagonal line indicates perfect calibration.

### Net Benefit

**Figure 3.**
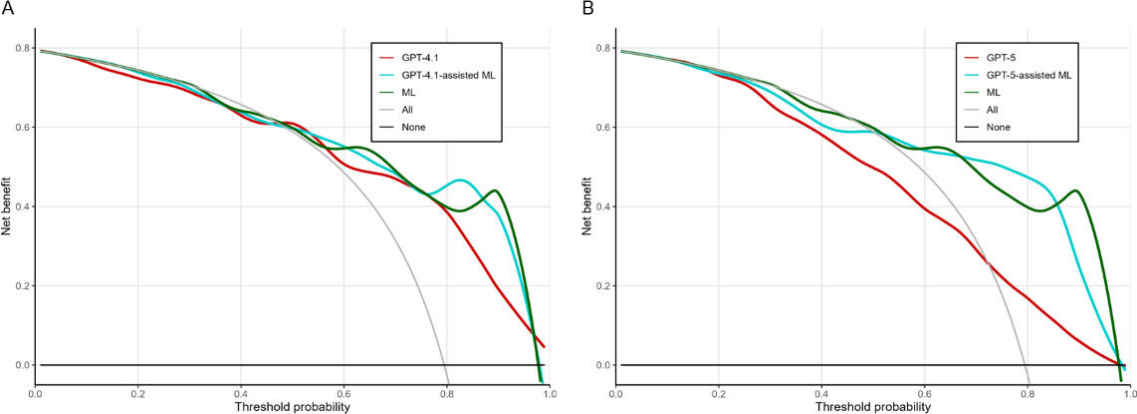
Decision curve analysis of GPT-4.1 and GPT-5 with and without model-informed prompting. ML: machine learning.

Figure 3 shows the results of the DCA, defining poor functional outcome as discharge mRS scores 3‐6. At threshold probabilities up to approximately 0.5, the net benefit was generally similar among the GPT-4.1 model, the ML-assisted GPT-4.1 model, the ML model, and the treat-all strategy (panel A). Specifically, at a threshold probability of 0.5, the net benefit was 0.61 (95% CI 0.51‐0.69) for the GPT-4.1 model and 0.60 (95% CI 0.50‐0.69) for the ML-assisted GPT-4.1 model, which were broadly comparable to that of the treat-all strategy (0.59, 95% CI 0.47‐0.69). At higher threshold probabilities, both the GPT-4.1 model and the ML-assisted GPT-4.1 model showed greater net benefit than the treat-all strategy. At a threshold probability of 0.8, the net benefit of the treat-all strategy was −0.03 (95% CI −0.31 to 0.23), whereas it was 0.35 (95% CI 0.27‐0.43) for the GPT-4.1 model and 0.49 (95% CI 0.37‐0.60) for the ML-assisted GPT-4.1 model.

Decision curves illustrate the relationship between the threshold probability and net benefit for GPT-4.1 (A) and GPT-5 (B). The red line represents the stand-alone GPT model without model-informed prompting, the blue line represents the ML-assisted GPT model incorporating outputs from the ML model, and the green line represents the ML-based model alone. The gray “All” line indicates the net benefit under the assumption that all patients experienced poor functional outcomes, whereas the black “None” line indicates the net benefit under the assumption that no patients experienced poor functional outcomes. Net benefit curves were estimated using LOESS smoothing to reduce variability and facilitate visual comparison across models. The vertical axis represents the net benefit, and the horizontal axis represents the threshold probability derived from each model’s predicted probability.

In contrast, for the GPT-5 model, the net benefit at a threshold probability of 0.5 was 0.50 (95% CI 0.42‐0.57), which appeared lower than that of the treat-all strategy (0.59, 95% CI 0.47‐0.69; panel B). For the ML-assisted GPT-5 model, the net benefit at the same threshold probability was 0.59 (95% CI 0.50‐0.67), which was broadly comparable to that of the treat-all strategy. At a threshold probability of 0.8, the net benefit was 0.16 (95% CI 0.11‐0.22) for the GPT-5 model and 0.46 (95% CI 0.38‐0.54) for the ML-assisted GPT-5 model; both of which appeared greater than that of the treat-all strategy. The ML-assisted GPT-5 model showed consistently higher net benefit than the GPT-5 model alone across the higher threshold range.

### Effects of Image Input on Discrimination and Volume Estimation

In the ablation analysis, the discriminative performance of the GPT-5 model using structured tabular clinical data alone yielded an AUROC of 0.79 (95% CI 0.71‐0.86; Multimedia Appendix 11). Discriminative performance improved with the addition of noncontrast head CT images, and the increase in AUROC was statistically significant based on 2000 bootstrap resamples (*P*=.02). Because the CT images were provided as JPEG files without spatial calibration or physical scale metadata, the volumes generated by GPT do not represent physically calibrated measurements but rather values inferred from visual proportions within the images. This methodological limitation should be considered when interpreting the results. To assess the extent to which GPT could approximate hematoma volume despite this constraint, we examined the correlation between GPT-derived estimates and volumes measured using the ABC/2 method. A strong positive monotonic association was observed between GPT-estimated hematoma volume and the 3D-measured hematoma volume obtained with the SYNAPSE VINCENT system (Spearman ρ=0.79, *P*<.001; Multimedia Appendix 12).

### Sensitivity Analysis in Patients Functionally Independent at Baseline

The analysis restricted to patients with premorbid mRS scores of 0‐1 produced overall patterns similar to those observed in the primary analysis, although the predictive performance was slightly reduced for all models (Multimedia Appendix 13). The FUNC score showed an AUROC of 0.75 (95% CI 0.68‐0.82). The conventional ML-based model achieved an AUROC of 0.80 (95% CI 0.72‐0.87), whereas GPT-4.1 and GPT-5 showed AUROCs of 0.79 (95% CI 0.71‐0.87) and 0.80 (95% CI 0.72‐0.87), respectively. For GPT-5, the scaled Brier score and Nagelkerke *R*² included negative values, indicating suboptimal calibration and overall predictive performance. Incorporating ML-derived information did not result in a clear improvement in discrimination; however, overall performance indices, including the scaled Brier score and Nagelkerke *R*², improved modestly. Calibration plots (Multimedia Appendix 14) showed that, consistent with the primary analysis, GPT models tended to underestimate predicted probabilities. The discrepancy between the observed and predicted probabilities was partially attenuated but not fully resolved after incorporating ML-derived information. In the DCA (Multimedia Appendix 15), similar to the primary analysis, the model-based strategies showed only limited net benefit, primarily in higher threshold ranges.

## Discussion

### Principal Results

In this study, even without task-specific training, both GPT-4.1 and GPT-5 demonstrated stable discrimination comparable to that of conventional ML models. Ablation analyses indicated that the inclusion of noncontrast head CT images significantly improved discriminative performance. However, GPT-based models alone exhibited limitations in calibration. Incorporating ML-derived outputs into GPT prompts yielded modest improvements in calibration, reproducibility, and decision-curve net benefit compared with stand-alone GPT models; however, these improvements did not translate into exceeding the performance of the conventional ML model.

### Prognostic Models Based on Specialist-Derived Variables

Several clinical scoring systems have been proposed to predict functional outcomes after ICH. Among them, the ICH FUNC score is one of the most widely used tools [19]. It estimates the probability of achieving functional independence—defined as a Glasgow Outcome Scale score ≥4—at 90 days after ICH occurrence based on the following 5 key factors: ICH volume, age, ICH location, Glasgow Coma Scale (GCS) score, and pre-ICH cognitive impairment. In our cohort, patients with poor outcomes showed unfavorable values across these components (Multimedia Appendix 16). Although the FUNC score demonstrated good predictive ability (AUROC 0.80, 95% CI 0.73‐0.86; Table 2) [4], its calculation requires specialist assessment, limiting its applicability in nonspecialist emergency settings.

To improve predictive precision and automation, several ML models have been developed using various variables, achieving AUROC values of 0.80 to 0.90 [4,6,20-29]. However, most models rely on expert-interpreted data, including GCS scoring and detailed image analysis, making them difficult to implement in EDs lacking specialist support. This limitation highlights the importance of investigating whether GPT models can achieve comparable predictive performance using only routinely available, nonspecialist data.

### Automated Prognostic Prediction Based on Nonspecialist Data

In recent years, automated approaches for predicting poststroke outcomes without reliance on specialist interpretation have been increasingly explored [6,20-24]. Among these, end-to-end deep learning models that optimize the entire pipeline—from image input to outcome prediction—without explicit region-of-interest extraction or manual feature engineering can streamline the inference process and support rapid decision-making under the time constraints of the ED. Indeed, end-to-end deep learning models using noncontrast CT images alone have reported discriminative performance with AUROC values of approximately 0.83 [6].

In our previous ML-based studies, we developed a fully automated deep learning preprocessing pipeline and achieved high predictive performance by integrating latent feature representations extracted from imaging data with routinely available clinical variables that can be obtained by nonspecialists in the ED [3,4]. However, in this framework, imaging information was encoded as latent feature vectors. Although the overall contribution of these image-derived features could be quantified using variable importance measures, intuitively understanding which specific anatomical regions or local imaging findings contributed to poor outcomes remained challenging. Thus, limitations in model interpretability persisted.

Against this background, this study explored an approach based on multimodal LLMs, focusing on their ability to convey the meaning of imaging findings and their clinical implications to clinicians through natural language interaction. As illustrated in Multimedia Appendix 1, this approach offers a degree of interpretability by enabling natural language explanations of imaging findings and their relevance to clinical decision-making. Even in a zero-shot setting without task-specific fine-tuning, GPT demonstrated discriminative performance within the range reported for widely used clinical scores such as the FUNC score. However, direct comparison was not feasible because of differences in outcome assessment timing, and therefore, these findings require cautious interpretation.

GPT-5 retained overall good discriminative performance but tended to slightly underestimate predicted probabilities relative to observed outcomes, indicating persistent challenges in calibration. The model with ML-derived information (such as predicted probabilities and regression coefficients) incorporated into the prompt demonstrated improved calibration compared with GPT alone; however, both the scaled Brier score and Nagelkerke *R*² of this model remained inferior to those of the stand-alone ML model. The latent features provided to the model were high-dimensional representations extracted by a deep learning U-Net, the semantic meaning of which is not directly interpretable by the LLM. Accordingly, explanations generated using these features should be regarded as post hoc rationalizations rather than true mechanistic interpretations and may represent synthetic clinical narratives. In addition, integrating the prior model’s predicted probability into the prompt likely introduced anchoring bias, whereby the LLM’s estimates are influenced by the supplied numerical value. This anchoring effect may partly explain the convergence between zero-shot and ML-assisted outputs and should be considered a key factor driving the observed performance changes. Routing predictions through GPT may reduce statistical calibration relative to the baseline model and produce a measurable trade-off between natural language interpretability and predictive reliability. This approach did not fully resolve the observed reliability limitations; further work is needed to develop robust guardrail mechanisms to ensure the safe clinical use of generative artificial intelligence systems.

The GPT-based pipeline evaluated in this study relies on a human-curated workflow, incorporating outputs from an ML model and manually selected representative CT slices, and, therefore, does not constitute a fully automated system for use in the ED. Rather than an end-to-end autonomous inference model, this approach should be viewed as a decision-support process that includes human curation. Although the required inputs do not involve assessments that can only be performed by stroke specialists, such as GCS scoring, it should be noted that effective slice selection still requires a certain level of radiological literacy. In addition, when GPT-5 was deployed via an API using the reasoning-minimal setting, inference required approximately 8.6 seconds per case, and token-based usage costs were incurred. In contrast, conventional ML models typically allow rapid inference with negligible per-case inference costs. Accordingly, careful consideration is required when applying large foundation models across all ED cases from an operational and cost perspective.

### Safety of GPT-Based Clinical Decision Support

The ablation analysis suggests that adding noncontrast head CT images provides information beyond structured tabular clinical data, contributing to prognostic inference. However, several considerations should be taken into account when interpreting these findings from a safety perspective. Previous studies on ICH subtype classification using noncontrast CT have reported that zero-shot multimodal LLMs, such as GPT-4o, Gemini 2.0 Flash, and Claude 3.5 Sonnet V2, do not achieve performance comparable to conventional deep learning models, including ResNet-50 and Vision Transformers, indicating persistent challenges in pixel-level image recognition [30]. In addition, the CT images provided to GPT in this study were in the JPEG format and did not explicitly encode physical scale information such as pixel spacing, slice thickness, or field of view. Consequently, absolute hematoma volume cannot be derived from these images. Within the ABC/2 framework, GPT-5 appears to rely on relatively coarse visual features—such as the longitudinal extent and overall morphology of the hematoma—to approximate relative hematoma burden, and these estimates show some correlation with reference measurements. Nevertheless, such estimates should be interpreted cautiously as imprecise and supplementary indicators rather than reliable quantitative measurements.

Beyond quantitative performance metrics, we conducted a brief qualitative case review focused on safety-relevant failure modes, particularly instances in which GPT produced high-confidence but incorrect predictions. Most errors were attributable to misinterpretation of CT imaging findings. In a false-positive thalamic hemorrhage case, the model assigned a high probability of mRS score 3‐6 at discharge despite an actual discharge mRS score of 1. The model appeared to interpret choroid plexus calcification within the lateral ventricle as hemorrhage and to infer ventricular extension, leading to risk overestimation. Conversely, in a false-negative case involving a large pontine hemorrhage, the model assigned a low probability of mRS score 3‐6 despite an actual discharge mRS score of 6. In this case, linear hyperattenuation caused by motion artifact reduced lesion conspicuity, resulting in underestimation of hemorrhage severity. These observations are consistent with previously reported limitations in the image-understanding capabilities of multimodal LLMs, in which judgment can become unstable under visual conditions that strongly depend on lesion contrast and visibility, as well as the presence of hyperattenuating structures or imaging artifacts [30]. Taken together, these findings underscore the importance of retaining human oversight and implementing safety guardrails when deploying multimodal LLMs in clinical decision-support settings.

### Utility in Clinical Decision Support

The clinical intervention scenario considered in this study involves decision support at ED arrival, specifically whether to initiate early transfer planning or to consider a home-discharge pathway for patients predicted to have poor functional outcomes at discharge (mRS score 3‐6). Patients with ICH often present with severe clinical features, which may lead clinicians to adopt pessimistic recovery expectations and favor intensive or transfer-oriented management strategies. Because accurately predicting functional outcomes (mRS ≥3) remains challenging in routine practice, precautionary decisions such as early transfer planning may be implemented even for patients who would ultimately achieve meaningful recovery. In this context, a prediction model could, in principle, help identify patients at relatively low risk of poor functional outcome who might be considered for home discharge rather than routine transfer-oriented care. However, the DCA for the present cohort of patients with acute ICH indicated that both the ML and GPT-based models provided net benefit only at relatively high decision thresholds compared with a treat-all strategy. Furthermore, the GPT-based models did not demonstrate a meaningful advantage in net benefit over the ML model, suggesting a limited incremental value in this setting. Restricting the analysis to patients who were functionally independent prior to onset did not materially alter these findings.

In this study, the prevalence of poor functional outcome approached 80%, resulting in a decision-curve pattern in which a treat-all strategy retained net benefit across a wide range of threshold probabilities. Consequently, the clinical utility of any predictive model in this setting is mathematically constrained to higher decision thresholds, because poor outcomes are overwhelmingly common and the default strategy already captures most events. From a decision-analytic perspective, net benefit depends not only on discrimination but also on model calibration [31]. The tendency toward risk underestimation observed in the stand-alone GPT models may therefore partly explain their reduced net benefit in certain threshold regions. Future research may benefit from developing prediction frameworks that jointly model outcome severity across multiple mRS strata (eg, mRS score 3‐4, mRS score 5‐6, and death). Such approaches could enable clearer differentiation of the clinical consequences of false-positive and false-negative predictions and may support more context-sensitive, interpretable, and clinically actionable decision support in emergency care settings.

### Limitations

This study had certain limitations. First, the dataset was derived from a single institution in Japan with a limited sample size, which may restrict the generalizability of the findings. Differences in the prevalence of hypertensive intracerebral hemorrhage and imaging acquisition protocols across institutions and regions may limit applicability to other populations. Second, this study focused on discharge mRS score as the prediction target, whereas many prior studies, including those using the FUNC score, evaluated functional outcomes at 90 days; therefore, differences in outcome timing introduce limitations in direct comparisons. Third, for multimodal inference, representative CT slices were manually selected by a radiologic technologist and converted into JPEG format, which may have introduced selection bias. Fourth, because single imputation was used instead of multiple imputation, some degree of bias may remain, even though the extent of missingness was minimal. Fifth, the ML pipeline used an L1-regularized logistic regression model, which is effective for variable selection but can produce biased coefficient estimates due to shrinkage; consequently, predicted probabilities derived from these coefficients may also be biased. Finally, for privacy protection, GPT-based inference should be performed within secure environments, such as Azure OpenAI, which imposes technical, financial, and governance constraints on clinical implementation.

### Conclusion

This study showed that zero-shot GPT models achieved discrimination comparable to that of a conventional ML model but did not demonstrate superior predictive performance. GPT-based models alone showed limitations in calibration, and routing predictions through GPT was associated with reduced statistical reliability, suggesting a trade-off between natural language interpretability and predictive robustness. Incorporating outputs from lightweight ML models into the prompts yielded modest improvements, although with remaining reliability concerns. The clinical usefulness of such systems appears to be context-dependent and may be restricted to higher decision-threshold regions in high-prevalence settings. Safe clinical deployment will likely require both system-level guardrails and human-in-the-loop oversight to ensure that final decisions remain under clinician control. These findings should also be interpreted in light of study-specific constraints, including nonautomated image input and inherent uncertainties in visual feature interpretation.

## Supplementary material

10.2196/87062 Multimedia Appendix 1 Example multimodal GPT-5 outputs using structured and computed tomography image data.

10.2196/87062 Multimedia Appendix 2 Patient selection for derivation and validation cohorts.

10.2196/87062 Multimedia Appendix 3 Acquisition parameters of computed tomography images.

10.2196/87062 Multimedia Appendix 4 Distribution of the number of representative computed tomography slices selected per patient.

10.2196/87062 Multimedia Appendix 5 Discriminative performance of GPT-5 measured by the area under the receiver operating characteristic curve and inference time.

10.2196/87062 Multimedia Appendix 6 Prompts used for GPT inference.

10.2196/87062 Multimedia Appendix 7 Standardized regression coefficients for the machine learning–based model.

10.2196/87062 Multimedia Appendix 8 Programs for data analysis.

10.2196/87062 Multimedia Appendix 9 Predictive performance and reproducibility of GPT-4.1 and GPT-5 with and without machine learning assistance.

10.2196/87062 Multimedia Appendix 10 Calibration plot of the machine learning–based model.

10.2196/87062 Multimedia Appendix 11 Discriminative performance of GPT-5 zero-shot models with and without imaging inputs.

10.2196/87062 Multimedia Appendix 12 Correlation of GPT-5–estimated and 3D-measured intracerebral hemorrhage volumes.

10.2196/87062 Multimedia Appendix 13 Predictive performance of GPT-based models, a machine learning–based model, and a clinical risk score in patients with premorbid modified Rankin Scale 0 to 1.

10.2196/87062 Multimedia Appendix 14 Calibration of the GPT-4.1 and GPT-5 models with and without model-informed prompting in patients with premorbid modified Rankin Scale 0 to 1.

10.2196/87062 Multimedia Appendix 15 Decision curve analysis of the GPT-4.1 and GPT-5 models with and without model-informed prompting in patients with premorbid modified Rankin Scale 0 to 1.

10.2196/87062 Multimedia Appendix 16 Differences in baseline clinical data according to functional outcome.

## Abbreviations

API: application programming interface
AUROC: area under the receiver operating characteristic curve
CT: computed tomography
DCA: decision curve analysis
DICOM: Digital Imaging and Communications in Medicine
ED: emergency department
GCS: Glasgow Coma Scale
ICC: intraclass correlation coefficient
ICH: intracerebral hemorrhage
LLM: large language model
LOESS: Locally Estimated Scatterplot Smoothing
ML: machine learning
mRS: modified Rankin Scale
NPV: negative predictive value
PPV: positive predictive value
TRIPOD: Transparent Reporting of a Multivariable Model for Individual Prognosis or Diagnosis
