# Supplementary material for: Multimodal GPT-5 for Predicting Poor Functional Outcomes After Intracerebral Hemorrhage in the Emergency Department: Validation Study
Source: JMIR AI. 2026 May 27;5:e87062. doi: 10.2196/87062 (PMC13216710; doi:10.2196/87062)
Supplement: Multimedia Appendix 1 [file ai-v5-e87062-s001.docx]

**
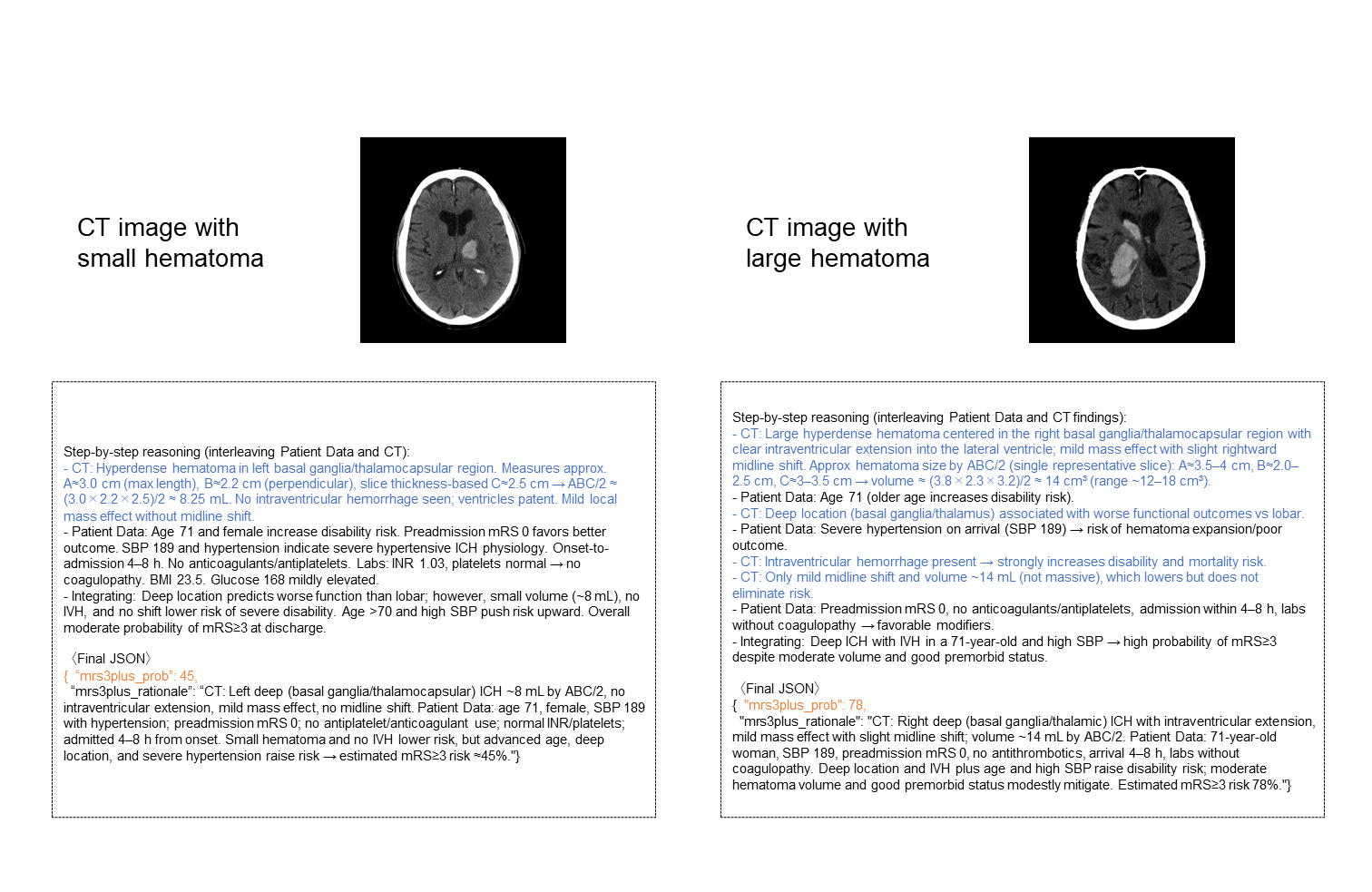
**
Multimedia Appendix 1. Example multimodal GPT-5 outputs using structured and CT image data

The pseudo patient data were identical between the two cases, whereas only the CT images differed in hematoma volume (A: small; B: large). Yellow and blue highlights indicate GPT-5's reasoning based on CT image interpretation and the corresponding predicted probabilities generated by GPT-5, respectively. The CT images were obtained from the publicly available Brain Hemorrhage Segmentation Dataset (BHSD) by Wu et al. and are licensed under the Creative Commons Attribution–NonCommercial–NoDerivatives 4.0 International (CC BY-NC-ND 4.0) license.
