# Supplementary figures and images for: Multimodal GPT-5 for Predicting Poor Functional Outcomes After Intracerebral Hemorrhage in the Emergency Department: Validation Study
Source: JMIR AI. 2026 May 27;5:e87062. doi: 10.2196/87062 (PMC13216710; doi:10.2196/87062)

**
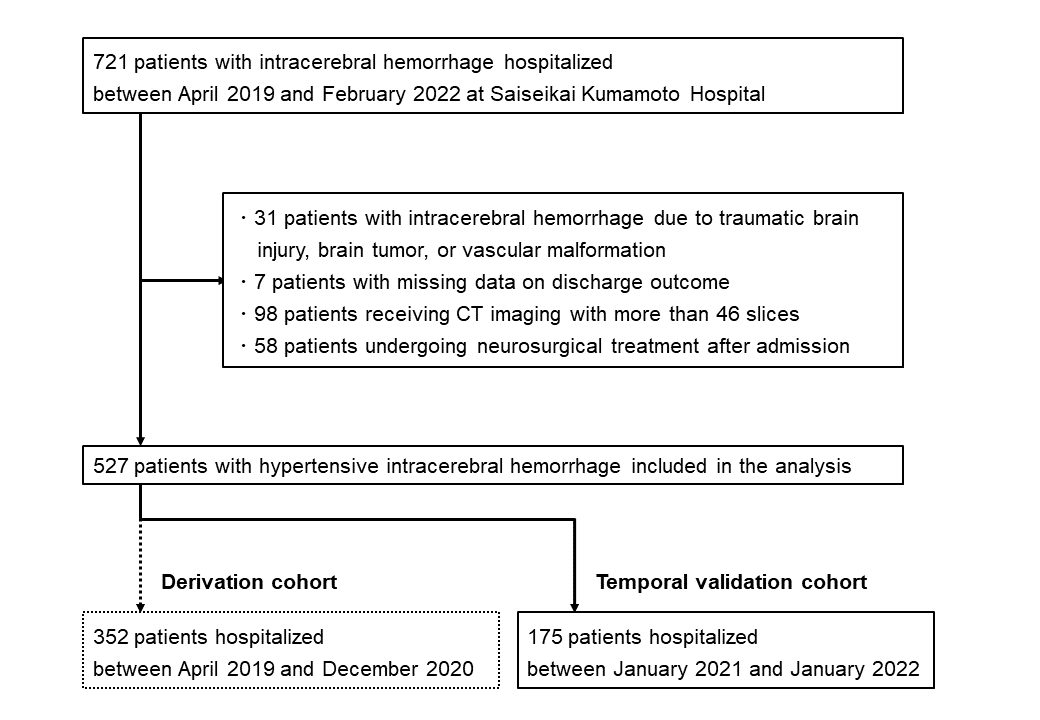
**

Multimedia Appendix 2. Patient selection for derivation and validation cohorts

Supplement: Multimedia Appendix 2 [file ai-v5-e87062-s002.docx]
