# Supplementary material for: Multimodal GPT-5 for Predicting Poor Functional Outcomes After Intracerebral Hemorrhage in the Emergency Department: Validation Study
Source: JMIR AI. 2026 May 27;5:e87062. doi: 10.2196/87062 (PMC13216710; doi:10.2196/87062)
Supplement: Multimedia Appendix 3 [file ai-v5-e87062-s003.docx]

Multimedia Appendix 3. Acquisition parameters of CT images

|  | Canon aquilion prime SP |
| --- | --- |
| Slice thickness, mm | 4.0 |
| Axial slice number | 14–45 |
| Voxel size, mm | 0.4844 × 0.4688 × 4.0 |
| Matrix size | 512 × 512 |
| Field of view, mm | 248 × 240 |
| Window width | 80 |
| Window level | 32 |

CT: computed tomography
