# Supplementary material for: Multimodal GPT-5 for Predicting Poor Functional Outcomes After Intracerebral Hemorrhage in the Emergency Department: Validation Study
Source: JMIR AI. 2026 May 27;5:e87062. doi: 10.2196/87062 (PMC13216710; doi:10.2196/87062)
Supplement: Multimedia Appendix 4 [file ai-v5-e87062-s004.docx]

**
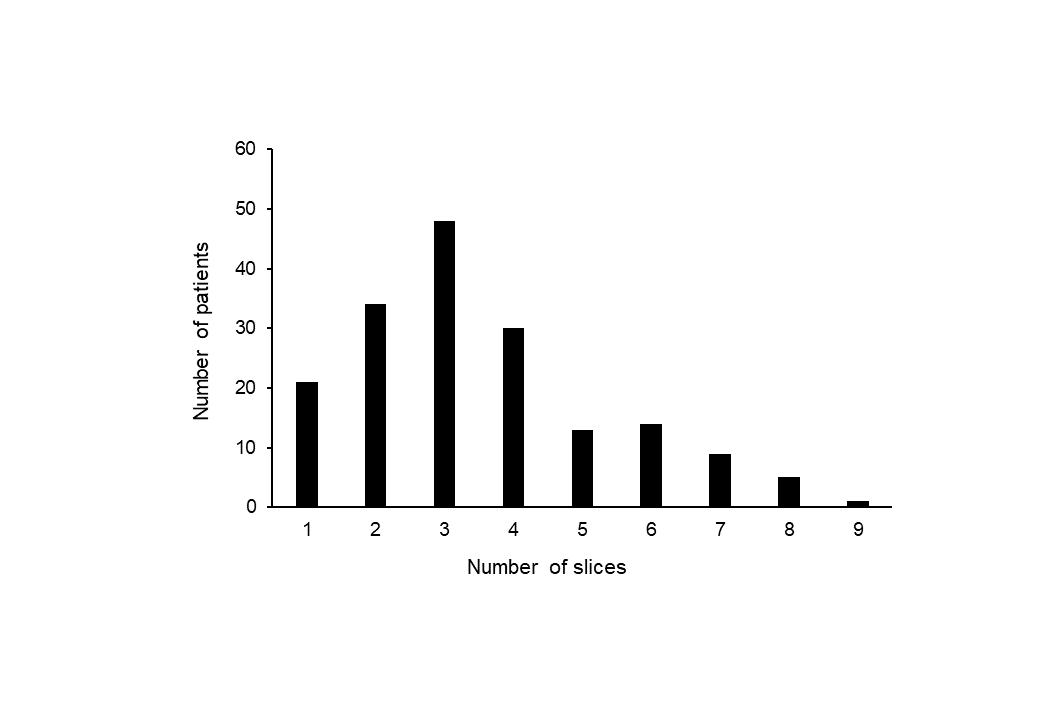
**

Multimedia Appendix 4. Distribution of the number of representative CT slices selected per patient

Horizontal axis indicates the number of CT slices selected per patient, and the vertical axis shows the number of patients. Representative noncontrast CT slices clearly demonstrating intracerebral hemorrhage were selected by a radiologic technologist according to predefined criteria and used for GPT inference.

CT: computed tomography
