# Supplementary material for: Multimodal GPT-5 for Predicting Poor Functional Outcomes After Intracerebral Hemorrhage in the Emergency Department: Validation Study
Source: JMIR AI. 2026 May 27;5:e87062. doi: 10.2196/87062 (PMC13216710; doi:10.2196/87062)
Supplement: Multimedia Appendix 5 [file ai-v5-e87062-s005.docx]

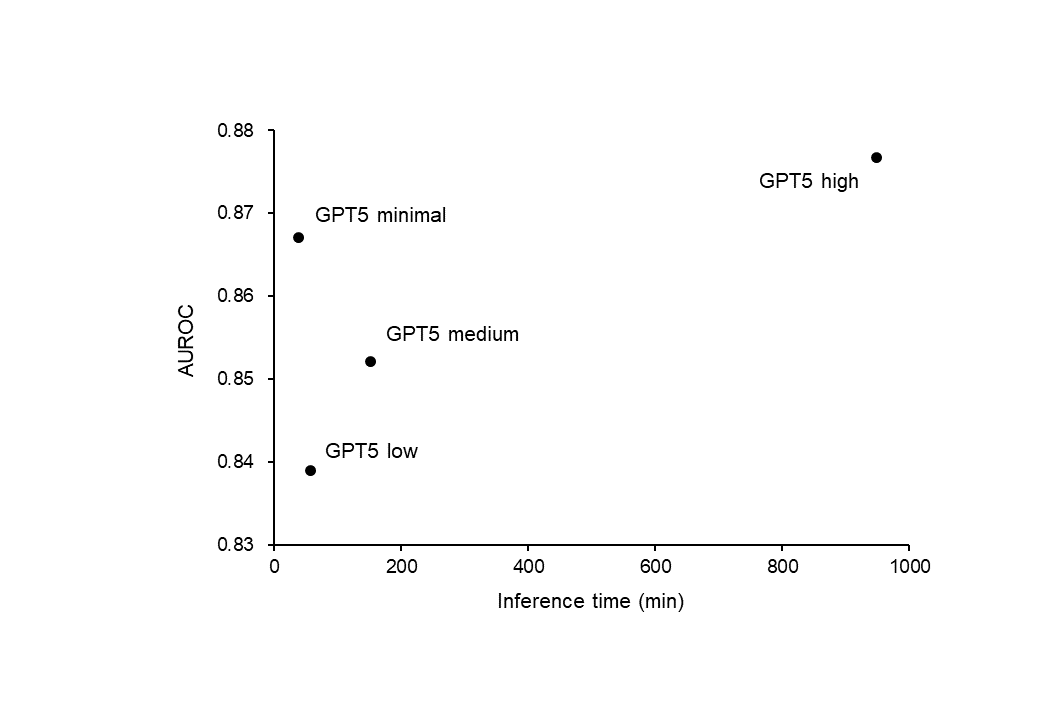


Multimedia Appendix 5. Discriminative performance of GPT-5 measured by the AUROC and inference time

The vertical axis represents the AUROC of GPT-5 inference based on 175 validation cases, while the horizontal axis shows the total inference time (in minutes) required to complete predictions for all 175 cases. These results highlight both the predictive accuracy and computational efficiency of GPT-5 when applied to real-world data.

AUROC: area under the receiver operating characteristic curve
