# Supplementary material for: Multimodal GPT-5 for Predicting Poor Functional Outcomes After Intracerebral Hemorrhage in the Emergency Department: Validation Study
Source: JMIR AI. 2026 May 27;5:e87062. doi: 10.2196/87062 (PMC13216710; doi:10.2196/87062)
Supplement: Multimedia Appendix 6 [file ai-v5-e87062-s006.docx]

**Multimedia Appendix 6. Prompts used for GPT inference.**

**Prompt S1. Tabular–image integration prompt**

*### system*

*You are a stroke specialist.*

*- You may use general medical knowledge, but base your reasoning only on the Patient Data and the attached noncontrast head CT image(s).*

*- Strictly follow the instructed output format. Avoid unnecessary words.*

*### attachment*

*Noncontrast head CT image file(s) named ".png" will be attached in a later step for integration.*

*### user*

*Using the **Patient Data below and the attached CT image(s)**, estimate the **risk (0–100)** that a patient admitted with intracerebral hemorrhage (ICH) will have a **discharge modified Rankin Scale (mRS) of 3 or greater**, and provide your rationale.*

*(Hereinafter, `mrs3plus` refers to discharge modified Rankin Scale ≥3.)*

*ID: ****

*Women: ****

*Use of ambulance: ****

*4–8 h from onset to admission: ****

*8–24 h from onset to admission: ****

*24–72 h from onset to admission: ****

*>72 h from onset to admission: ****

*Body mass index: ****

*Preadmission mRS: ****

*Age: ****

*Taking antiplatelet drugs: ****

*Taking direct oral anticoagulants (DOACs): ****

*Taking warfarin: ****

*Diabetes mellitus: ****

*Dyslipidemia: ****

*Hypertension: ****

*Smoking: ****

*Drinking: ****

*vital sign_SBP: ****

*vital sign_DBP: ****

*vital sign_PR: ****

*vital sign_SpO_2_: ****

*vital sign_RR: ****

*vital sign_BT: ****

*blood test_Na: ****

*blood test_K: ****

*blood test_Cl: ****

*blood test_TP: ****

*blood test_ALB: ****

*blood test_BUN: ****

*blood test_CRE: ****

*blood test_AST: ****

*blood test_ALT: ****

*blood test_γ GTP: ****

*blood test_LD: ****

*blood test_T Bil: ****

*blood test_glucose: ****

*blood test_WBC: ****

*blood test_RBC: ****

*blood test_hemoglobin: ****

*blood test_hematocrit: ****

*blood test_BPC: ****

*blood test_CRP: ****

*blood test_APTT: ****

*blood test_PT INR: ****

***Requirements***

*1) Provide a **step-by-step reasoning (≤1200 characters)** in English that **interleaves**:*

*- key points from the **Patient Data**, and*

*- explicit **CT image findings** (e.g., hemorrhage location, intraventricular extension, mass effect/midline shift, approximate hematoma volume using ABC/2 if feasible).*

*Make it clear which parts come from the Patient Data and which come from the image(s).*

*2) Then output **〈Final JSON〉** containing only the keys below.*

*【JSON Keys】*

*- "mrs3plus_prob" : integer from 0 to 100*

*- "mrs3plus_rationale" : concise rationale that **explicitly cites both** patient data and image findings*

**Prompt S2. Model-informed prompt**

*### system*

*You are a stroke specialist.*

*- Use general medical knowledge, but ground your reasoning only in the Patient Data,*

*the attached noncontrast head-CT image(s), and the Prior Model information below.*

*- Follow the output format exactly; omit unnecessary words.*

*### prior-model*

*The **Prior Model** is a late-fusion model that combines deep-learning image features*

*predictive of poor functional outcome (discharge mRS ≥ 3) with tabular clinical data*

*using L1-penalized logistic regression (LASSO).*

*Prior Model Prediction (mrs3plus_prob): ***%*

*Model Coefficients (standardized):*

*- Image features extracted by deep learning 26: +1.0399*

*- Pre-stroke mRS: +0.5191*

*- Use of ambulance: +0.2434*

*- Laboratory data_Lactate dehydrogenase, IU/L: +0.1564*

*- Image features extracted by deep learning 425: +0.0038*

*- Image features extracted by deep learning 397: –0.0217*

*- Image features extracted by deep learning 185: –0.0541*

*- Laboratory data_Sodium, mmol/L: –0.0695*

*- Laboratory data_Red blood cell count, 106/μL: –0.1890*

*- Drinking: -0.1943*

*- Onset-to-admission time >72 h: –0.2063*

*- Onset-to-admission time 24–72 h: -0.3352*

*- Image features extracted by deep learning 394: –0.4518*

*- Image features extracted by deep learning 371: –0.4784*

*Note:*

*- The **Patient Data** block below also includes the **deep-learning image feature*

*values (by feature ID)** used by the Prior Model for this case (e.g., "Image features*

*extracted by deep learning 26: …"). Treat these as the observed feature values for*

*this patient and use them to justify any upward/downward adjustment relative to the*

*prior probability.*

*### attachment*

*Noncontrast head-CT image file(s): ".png"*

*###user*

*Using the **Patient Data**, the **attached CT image(s)**, and the **Prior Model*

*information**, estimate the **risk of discharge mRS ≥ 3 (0–100)** for*

*intracerebral hemorrhage (ICH) and explain your reasoning.*

*ID: ****

*Women: ****

*Use of ambulance: ****

*4–8 h from onset to admission: ****

*8–24 h from onset to admission: ****

*24–72 h from onset to admission: ****

*>72 h from onset to admission: ****

*Body mass index: ****

*Preadmission mRS: ****

*Age: ****

*Taking antiplatelet drugs: ****

*Taking direct oral anticoagulants (DOACs): ****

*Taking warfarin: ****

*Diabetes mellitus: ****

*Dyslipidemia: ****

*Hypertension: ****

*Smoking: ****

*Drinking: ****

*vital sign_SBP: ****

*vital sign_DBP: ****

*vital sign_PR: ****

*vital sign_SpO_2_: ****

*vital sign_RR: ****

*vital sign_BT: ****

*blood test_Na: ****

*blood test_K: ****

*blood test_Cl: ****

*blood test_TP: ****

*blood test_ALB: ****

*blood test_BUN: ****

*blood test_CRE: ****

*blood test_AST: ****

*blood test_ALT: ****

*blood test_γ GTP: ****

*blood test_LD: ****

*blood test_T Bil: ****

*blood test_glucose: ****

*blood test_WBC: ****

*blood test_RBC: ****

*blood test_hemoglobin: ****

*blood test_hematocrit: ****

*blood test_BPC: ****

*blood test_CRP: ****

*blood test_APTT: ****

*blood test_PT INR: ****

*Image features extracted by deep learning 26: ****

*Image features extracted by deep learning 185: ****

*Image features extracted by deep learning 371: ****

*Image features extracted by deep learning 394: ****

*Image features extracted by deep learning 397: ****

*Image features extracted by deep learning 425: ****

***Requirements***

*1) Provide **step-by-step reasoning (≤1200 characters, English)** that interleaves:*

*• key points from **Patient Data***

*• explicit **CT findings** (location, IVH, mass effect, ABC/2 volume, etc.)*

*• how the **Prior Model prediction & coefficients** guide your adjustment*

*2) Then output **〈Final JSON〉** containing *only* the keys below.*

*【JSON Keys】*

*- "mrs3plus_prob" : integer 0–100*

*- "mrs3plus_rationale" : concise rationale citing patient data, image findings,*

*and prior model*
