# Supplementary material for: Multimodal GPT-5 for Predicting Poor Functional Outcomes After Intracerebral Hemorrhage in the Emergency Department: Validation Study
Source: JMIR AI. 2026 May 27;5:e87062. doi: 10.2196/87062 (PMC13216710; doi:10.2196/87062)
Supplement: Multimedia Appendix 7 [file ai-v5-e87062-s007.docx]

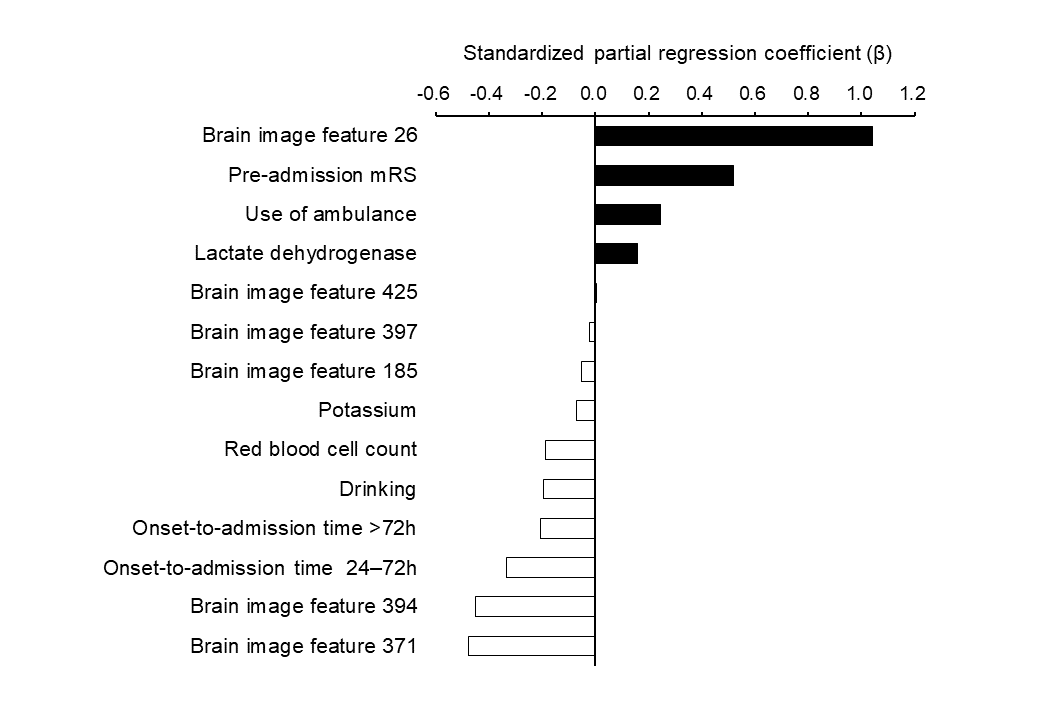


Multimedia Appendix 7. Standardized regression coefficients for the ML-based model

Variable importance was assessed based on the standardized partial regression coefficients (β) from the L1 regularized logistic regression models. Positive coefficients show an increased risk of poor functional outcome, while negative coefficients indicate a decreased risk.

ML: machine learning
