# Supplementary material for: Multimodal GPT-5 for Predicting Poor Functional Outcomes After Intracerebral Hemorrhage in the Emergency Department: Validation Study
Source: JMIR AI. 2026 May 27;5:e87062. doi: 10.2196/87062 (PMC13216710; doi:10.2196/87062)
Supplement: Multimedia Appendix 8 [file ai-v5-e87062-s008.docx]

## Multimedia Appendix 8. Programs for data analysis

### Programs for data analysis

Prompt generation for 175 participants was performed in Python (version 3.13.7). Inference with GPT-5 via the Azure OpenAI API was conducted in Python (version 3.13.7), with code available in the GitHub repository (https://github.com/kkmmmmm/GPT-zero-shot-inference/tree/main). Statistical analyses and metric calculation were performed using R Studio (version 2025.05.0) and R (version 4.5.0), respectively. The pROC package (https://cran.r-project.org/web/packages/pROC/pROC.pdf) was used to calculate the AUROC, sensitivity, specificity, positive predictive value, and negative predictive value. Intraclass correlation coefficients were calculated using the irr package (https://cran.r-project.org/web/packages/irr/irr.pdf). Decision curve analysis was performed using the rmda package (https://cran.r-project.org/package=rmda

). Calibration plots were generated using locally estimated scatterplot smoothing (LOESS; stats::loess) with patient-level bootstrap resampling implemented via custom R functions.
