# Supplementary material for: Multimodal GPT-5 for Predicting Poor Functional Outcomes After Intracerebral Hemorrhage in the Emergency Department: Validation Study
Source: JMIR AI. 2026 May 27;5:e87062. doi: 10.2196/87062 (PMC13216710; doi:10.2196/87062)
Supplement: Multimedia Appendix 9 [file ai-v5-e87062-s009.docx]

Multimedia Appendix 9. Predictive performance and reproducibility of GPT-4.1 and GPT-5 with and without ML assistance

|  | AUROC | Sensitivity | Specificity | PPV | NPV | ICC |
| --- | --- | --- | --- | --- | --- | --- |
| **Zero-shot model** |  |  |  |  |  |  |
| GPT-4.1 | 0.83 (0.01) | 0.61 (0.03) | 0.93 (0.02) | 0.97 (0.01) | 0.38 (0.02) | 0.91 (0.89–0.93) |
| GPT-5 | 0.86 (0.01) | 0.72 (0.08) | 0.88 (0.07) | 0.96 (0.02) | 0.46 (0.05) | 0.95 (0.94–0.96) |
| **ML-assisted model** |  |  |  |  |  |  |
| GPT-4.1-assisted by ML | 0.85 (0.01) | 0.71 (0.01) | 0.90 (0.02) | 0.97 (0.01) | 0.45 (0.01) | 0.97 (0.96–0.98) |
| GPT-5-assisted by ML | 0.87 (0.00) | 0.65 (0.05) | 0.98 (0.02) | 0.99 (0.01) | 0.42 (0.03) | 0.96 (0.95–0.97) |

AUROC: area under the receiver operating characteristic curve, PPV: positive predictive value, NPV: negative predictive value, ICC: intraclass correlation coefficient, ML: machine learning.

For GPT-4.1 and GPT-5, the AUROC, sensitivity, specificity, PPV, and NPV are reported as the mean values across five inference runs, with the standard deviation shown in parentheses. Under the minimal reasoning-effort setting, inference was repeated five times per patient for both models, and the ICC was estimated using a single-score, one-way random-effects model. ICC values are reported as point estimates with 95% confidence intervals.
