# Supplementary material for: Multimodal GPT-5 for Predicting Poor Functional Outcomes After Intracerebral Hemorrhage in the Emergency Department: Validation Study
Source: JMIR AI. 2026 May 27;5:e87062. doi: 10.2196/87062 (PMC13216710; doi:10.2196/87062)
Supplement: Multimedia Appendix 10 [file ai-v5-e87062-s010.docx]

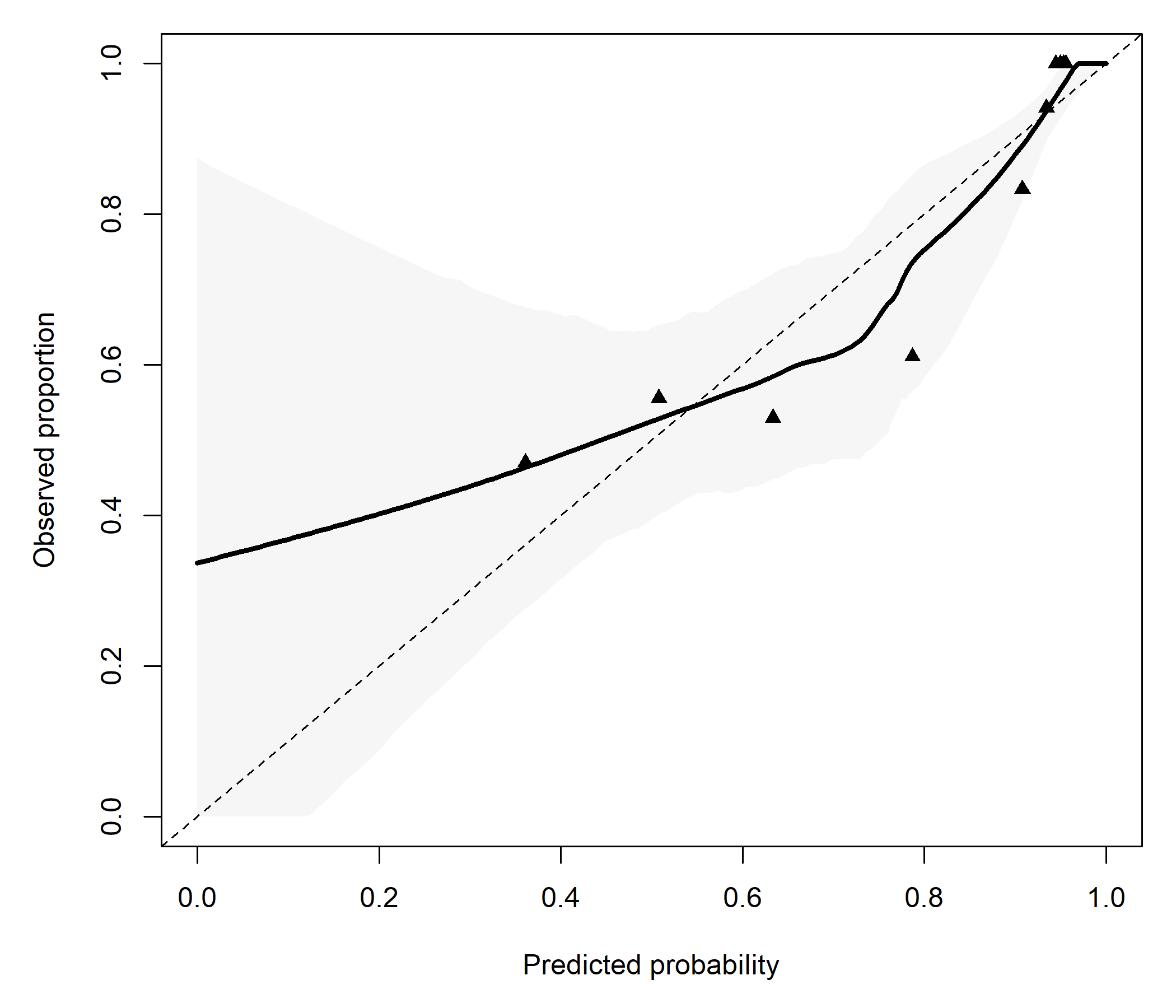


Multimedia Appendix 10. Calibration plot of the ML-based model

Patients were stratified into 10 groups based on the predicted probability of poor functional outcome in the validation cohort. For each risk group, the mean predicted probability (x-axis) was plotted against the observed event rate (y-axis). A loess-smoothed calibration curve with a pointwise 95% confidence interval was overlaid; the dashed diagonal line indicates perfect calibration.

ML: machine learning
