# Supplementary material for: Multimodal GPT-5 for Predicting Poor Functional Outcomes After Intracerebral Hemorrhage in the Emergency Department: Validation Study
Source: JMIR AI. 2026 May 27;5:e87062. doi: 10.2196/87062 (PMC13216710; doi:10.2196/87062)
Supplement: Multimedia Appendix 11 [file ai-v5-e87062-s011.docx]

Multimedia Appendix 11. Discriminative performance of GPT-5 zero-shot models with and without imaging inputs

|  | AUROC | Sensitivity | Specificity | PPV | NPV |
| --- | --- | --- | --- | --- | --- |
| **Zero-shot model** |  |  |  |  |  |
| GPT-5 (tabular only) | 0.79 (0.71–0.86) | 0.59 (0.42–0.85) | 0.89 (0.64–1.00) | 0.95 (0.89–1.00) | 0.36 (0.27–0.53) |
| GPT-5 (tabular + image) | 0.85 (0.78–0.91) | 0.68 (0.58–0.85) | 0.92 (0.76–1.00) | 0.97 (0.92–1.00) | 0.42 (0.32–0.60) |

AUROC: area under the receiver operating characteristic curve, PPV: positive predictive value, NPV: negative predictive value.

GPT-5 (tabular only) indicates inference performed using structured tabular clinical data alone, whereas GPT-5 (tabular + image) indicates inference performed using structured tabular clinical data in combination with noncontrast head CT images. Values are presented as point estimates with 95% confidence intervals in parentheses. All 95% confidence intervals were estimated using 2,000 bootstrap resamples.
