# Supplementary material for: Multimodal GPT-5 for Predicting Poor Functional Outcomes After Intracerebral Hemorrhage in the Emergency Department: Validation Study
Source: JMIR AI. 2026 May 27;5:e87062. doi: 10.2196/87062 (PMC13216710; doi:10.2196/87062)
Supplement: Multimedia Appendix 12 [file ai-v5-e87062-s012.docx]

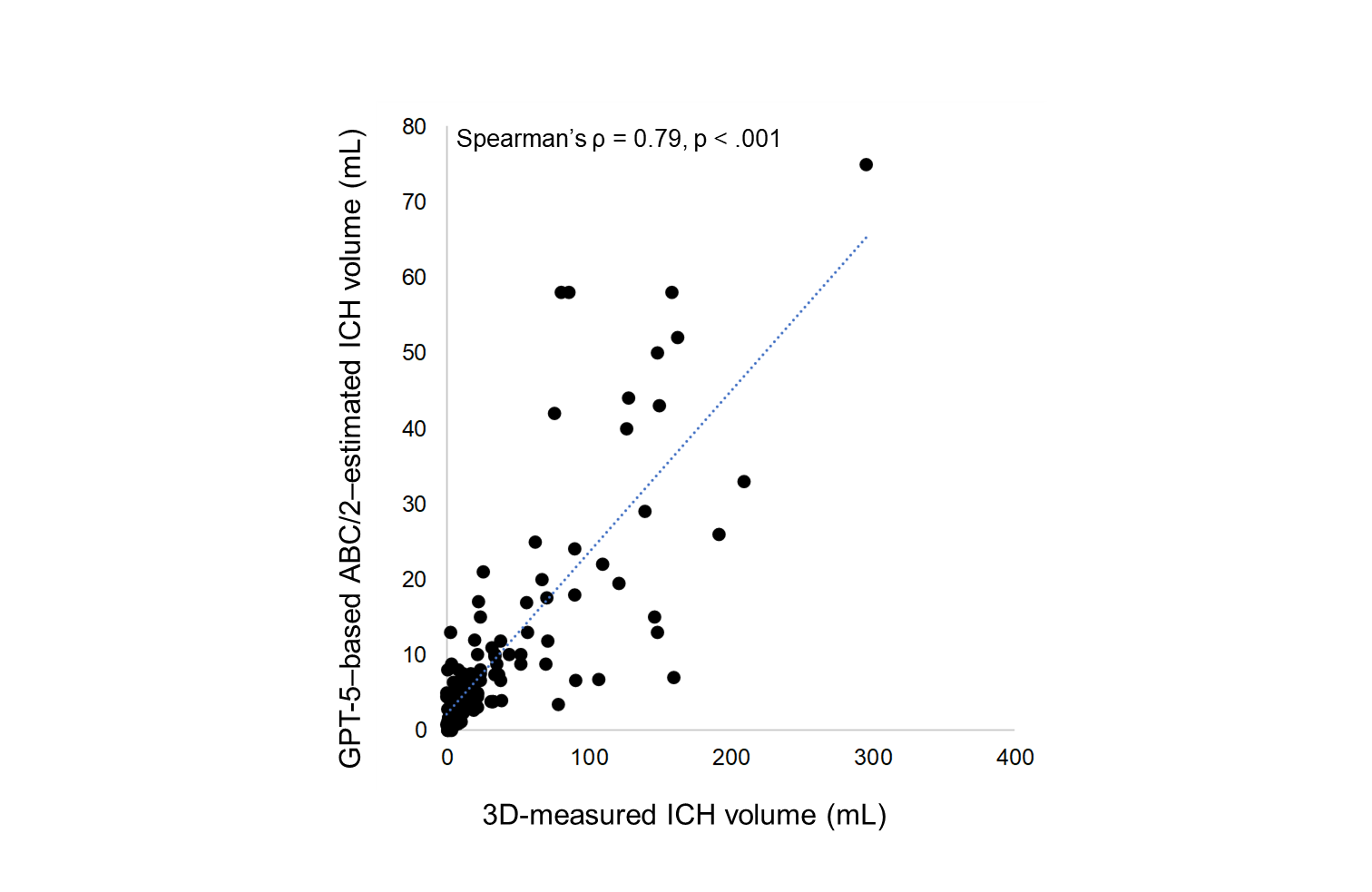


Multimedia Appendix 12. Correlation of GPT-5–Estimated and 3D-Measured ICH Volumes

Each dot represents an individual patient. The x-axis shows intracerebral hemorrhage (ICH) volume measured by 3D volumetric analysis, and the y-axis shows ICH volume estimated by GPT-5 using the ABC/2 method. A strong positive monotonic association was observed (Spearman’s ρ = 0.79, p < .001). The dashed line indicates the fitted linear trend.
