# Supplementary material for: Multimodal GPT-5 for Predicting Poor Functional Outcomes After Intracerebral Hemorrhage in the Emergency Department: Validation Study
Source: JMIR AI. 2026 May 27;5:e87062. doi: 10.2196/87062 (PMC13216710; doi:10.2196/87062)
Supplement: Multimedia Appendix 13 [file ai-v5-e87062-s013.docx]

Multimedia Appendix 13. Predictive performance of the GPT- and ML-based models and a clinical risk score in patients with premorbid mRS 0 to 1

|  | AUROC | Sensitivity | Specificity | PPV | NPV | Scaled BS | Nagelkerke’s R² |
| --- | --- | --- | --- | --- | --- | --- | --- |
| **Risk score** |  |  |  |  |  |  |  |
| FUNC score | 0.75 (0.68 to 0.82) | 0.54 (0.42 to 0.65) | 0.94 (0.86 to 1.00) | 0.96 (0.91 to 1.00) | 0.43 (0.32 to 0.54) |  |  |
| **Model Type** |  |  |  |  |  |  |  |
| ML-based model | 0.80 (0.72 to 0.87) | 0.60 (0.50 to 0.84) | 0.94 (0.71 to 1.00) | 0.97 (0.88 to 1.00) | 0.46 (0.36 to 0.65) | 0.17 (0.01 to 0.31) | 0.27 (0.06 to 0.42) |
| **Zero-shot model** |  |  |  |  |  |  |  |
| GPT-4.1 | 0.79 (0.71 to 0.87) | 0.78 (0.43 to 0.85) | 0.69 (0.63 to 1.00) | 0.88 (0.84 to 1.00) | 0.53 (0.34 to 0.68) | 0.08 (–0.20 to 0.27) | 0.11 (–0.26 to 0.35) |
| GPT-5 | 0.80 (0.72 to 0.87) | 0.56 (0.48 to 0.84) | 0.92 (0.67 to 1.00) | 0.95 (0.86 to 1.00) | 0.43 (0.34 to 0.64) | –0.18 (-  –0.58 to 0.07) | –0.22 (–0.77 to 0.10) |
| **ML-assisted model** |  |  |  |  |  |  |  |
| GPT-4.1-assisted by ML | 0.79 (0.71 to 0.86) | 0.60 (0.51 to 0.85) | 0.92 (0.67 to 1.00) | 0.95 (0.86 to 1.00) | 0.45 (0.34 to 0.64) | 0.16 (–0.04 to 0.32) | 0.26 (0.01 to 0.43) |
| GPT-5-assisted by ML | 0.82 (0.75 to 0.88) | 0.61 (0.49 to 0.78) | 0.94 (0.81 to 1.00) | 0.97 (0.91 to 1.00) | 0.47 (0.36 to 0.62) | 0.14 (–0.14 to 0.31) | 0.22 (–0.09 to 0.41) |

Values are presented as point estimates with 95% confidence intervals in parentheses. All 95% confidence intervals were estimated using 2,000 bootstrap resamples.

AUROC: area under the receiver operating characteristic curve, BS: Brier score, ML: machine learning, mRS: modified Rankin Scale, NPV: negative predictive value, PPV: positive predictive value.
