# Supplementary material for: Multimodal GPT-5 for Predicting Poor Functional Outcomes After Intracerebral Hemorrhage in the Emergency Department: Validation Study
Source: JMIR AI. 2026 May 27;5:e87062. doi: 10.2196/87062 (PMC13216710; doi:10.2196/87062)
Supplement: Multimedia Appendix 14 [file ai-v5-e87062-s014.docx]

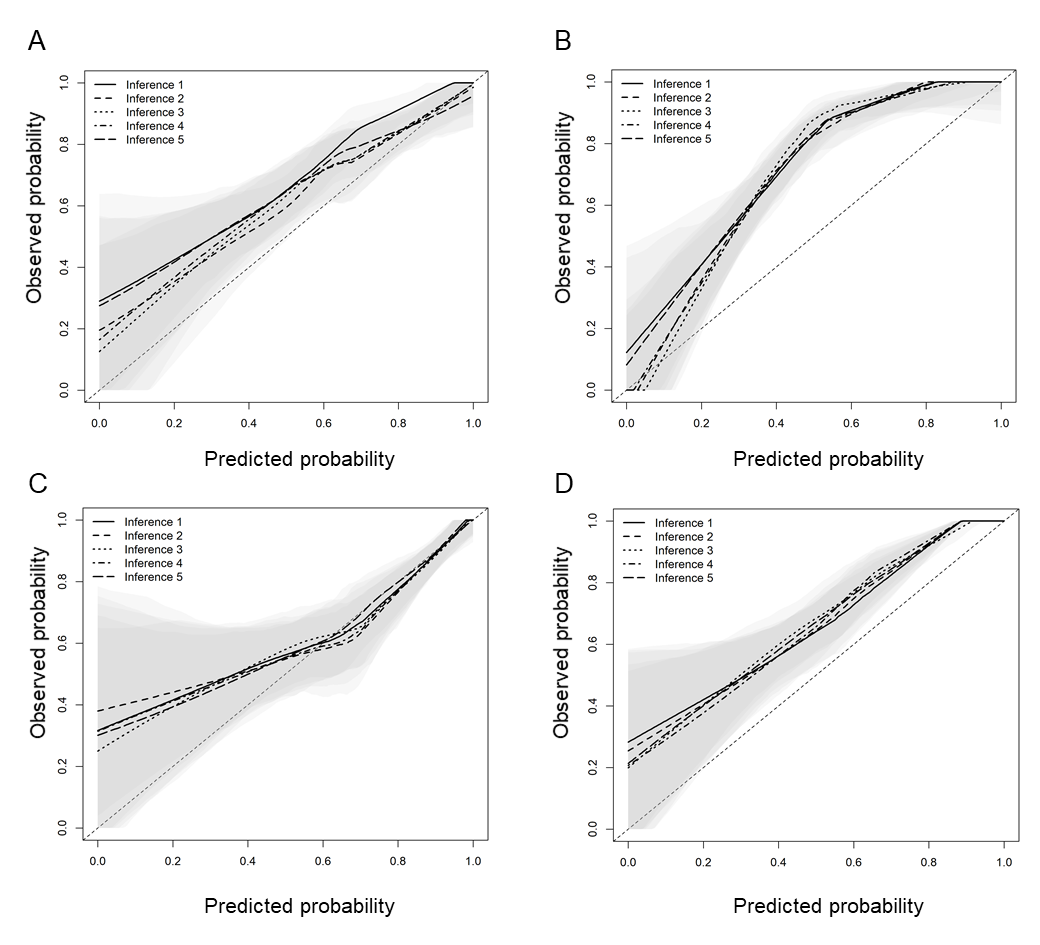


Multimedia Appendix 14. Calibration of the GPT-4.1 and GPT-5 models with and without model-informed prompting in patients with premorbid mRS 0 to 1.

Calibration plots for (A) GPT-4.1, (B) GPT-5, (C) model-informed GPT-4.1, and (D) model-informed GPT-5 models in the validation cohort. The relationship between predicted probabilities (x-axis) and observed probabilities (y-axis) is shown using a loess-smoothed calibration curve. The shaded area represents the pointwise 95% confidence interval estimated by patient-level bootstrap resampling. Predicted probabilities from five independent inference runs are overlaid to illustrate inter-run variability and reproducibility. The dashed diagonal line indicates perfect calibration.
