# Supplementary material for: Multimodal GPT-5 for Predicting Poor Functional Outcomes After Intracerebral Hemorrhage in the Emergency Department: Validation Study
Source: JMIR AI. 2026 May 27;5:e87062. doi: 10.2196/87062 (PMC13216710; doi:10.2196/87062)
Supplement: Multimedia Appendix 15 [file ai-v5-e87062-s015.docx]

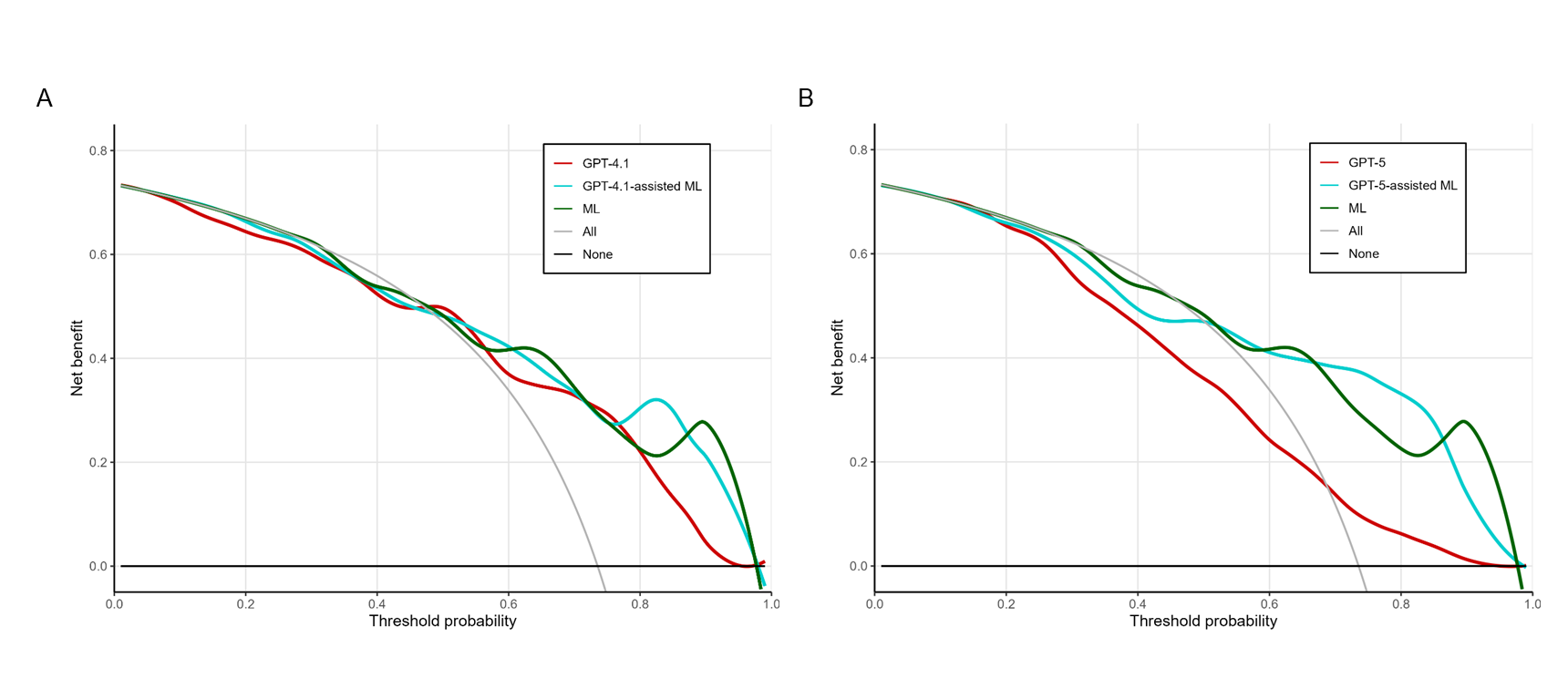


Multimedia Appendix 15. Decision curve analysis of the GPT-4.1 and GPT-5 models with and without model-informed prompting in patients with premorbid mRS 0 to 1.

Decision curves illustrate the relationship between the threshold probability and net benefit for (A) GPT-4.1 and (B) GPT-5 models. The red line represents the standalone GPT model without model-informed prompting, the blue line represents the ML-assisted GPT model incorporating outputs from the ML model, and the green line represents the ML-based model alone. The gray “All” line indicates the net benefit under the assumption that all patients experienced poor functional outcomes, whereas the black “None” line indicates the net benefit under the assumption that no patients experienced poor functional outcomes. Net benefit curves were estimated using loess smoothing to reduce variability and facilitate visual comparison across models. The vertical axis represents the net benefit, and the horizontal axis represents the threshold probability derived from each model’s predicted probability.
