# Supplementary material for: Multimodal GPT-5 for Predicting Poor Functional Outcomes After Intracerebral Hemorrhage in the Emergency Department: Validation Study
Source: JMIR AI. 2026 May 27;5:e87062. doi: 10.2196/87062 (PMC13216710; doi:10.2196/87062)
Supplement: Multimedia Appendix 16 [file ai-v5-e87062-s016.docx]

Multimedia Appendix 16. Differences in baseline clinical data according to functional outcome

|  | mRS 0–2 | mRS 3–6 | P-value |
| --- | --- | --- | --- |
| Demographic |  |  |  |
| Age, y | 67.5 (55.3–73.5) | 77 (66.5–85) | <0.001 |
| Female | 9 (25.0) | 60 (43.2) | 0.072 |
| Risk factor |  |  |  |
| Hypertension | 27 (75.0) | 93 (69.9) | 0.698 |
| Diabetes mellitus | 11 (30.6) | 26 (19.5) | 0.234 |
| Dyslipidemia | 11 (30.6) | 41 (30.8) | 1.000 |
| Smoking | 19 (52.8) | 41 (29.7) | 0.017 |
| Drinking | 15 (44.1) | 54 (39.7) | 0.785 |
| Pre-stroke functional status |  |  |  |
| Pre-stroke mRS score | 0 (0–0) | 0 (0–2) | 0.001 |
| Onset-to-admission time |  |  | 0.247 |
| <4 h | 19 (52.8) | 72 (51.8) |  |
| 4–8 h | 5 (13.9) | 13 (9.4) |  |
| 8–24 h | 7 (19.4) | 32 (23.0) |  |
| 24–72 h | 3 (8.3) | 21 (15.1) |  |
| >72 h | 2 (5.6) | 1 (0.7) |  |
| Glasgow Coma Scale score | 15 (15–15) | 14 (9–15) | <0.001 |
| Brain image finding |  |  |  |
| ICH volume, mL | 3.6 (1.8–8.0) | 12.6 (6.0–38.0) | <0.001 |
| ICH location |  |  | 0.515 |
| Thalamus | 8 (22.2) | 42 (30.2) |  |
| Putamen | 15 (41.7) | 39 (28.1) |  |
| Subcortex | 8 (22.2) | 33 (23.7) |  |
| Brainstem and pons | 1 (2.8) | 12 (8.6) |  |
| Cerebellum | 3 (8.3) | 7 (5.0) |  |
| Others | 1 (2.8) | 6 (4.3) |  |

mRS: modified Rankin Scale, ICH: intracerebral hemorrhage, ED: emergency department

Data are expressed as median (interquartile range) or n (%). ICH volume was measured using the Fujifilm Medical SYNAPSE VINCENT 3D image analysis system. Variables collected after the ED evaluation, as well as those requiring specialist interpretation for conventional risk score calculation, were excluded from the multimodal models. These data are presented solely to compare the patients' baseline characteristics according to functional outcomes.
